# Hotspot site microenvironment in the deubiquitinase OTUB1 drives its stability and aggregation

**DOI:** 10.1016/j.jbc.2024.107315

**Published:** 2024-04-23

**Authors:** Sushanta Majumder, Mitul Srivastava, Parvez Alam, Sandhini Saha, Raniki Kumari, Ajay Kumar Chand, Shailendra Asthana, Sobhan Sen, Tushar Kanti Maiti

**Affiliations:** 1 Functional Proteomics Laboratory, Regional Centre for Biotechnology, NCR Biotech Science Cluster, Faridabad, India; 2 Translational Health Science and Technology Institute, NCR Biotech Science Cluster, Faridabad, India; 3 Spectroscopy Laboratory, School of Physical Sciences, Jawaharlal Nehru University, New Delhi, India

**Keywords:** protein aggregation, protein misfolding disease, Parkinson's disease, lewy body, deubiquitinase, OTUB1, heterotypic aggregation

## Abstract

Lewy bodies (LB) are aberrant protein accumulations observed in the brain cells of individuals affected by Parkinson's disease (PD). A comprehensive analysis of LB proteome identified over a hundred proteins, many co-enriched with α-synuclein, a major constituent of LB. Within this context, OTUB1, a deubiquitinase detected in LB, exhibits amyloidogenic properties, yet the mechanisms underlying its aggregation remain elusive. In this study, we identify two critical sites in OTUB1—namely, positions 133 and 173—that significantly impact its amyloid aggregation. Substituting alanine at position 133 and lysine at position 173 enhances both thermodynamic and kinetic stability, effectively preventing amyloid aggregation. Remarkably, lysine at position 173 demonstrates the highest stability without compromising enzymatic activity. The increased stability and inhibition of amyloid aggregation are attributed mainly to the changes in the specific microenvironment at the hotspot. In our exploration of the *in-vivo* co-occurrence of α-synuclein and OTUB1 in LB, we observed a synergistic modulation of each other’s aggregation. Collectively, our study unveils the molecular determinants influencing OTUB1 aggregation, shedding light on the role of specific residues in modulating aggregation kinetics and structural transition. These findings contribute valuable insights into the complex interplay of amino acid properties and protein aggregation, with potential implications for understanding broader aspects of protein folding and aggregation phenomena.

Protein aggregation is a generic event in many neurodegenerative conditions, such as Alzheimer’s disease (AD) and Parkinson’s disease (PD). The initiation of protein aggregation involves the formation of partially unfolded conformers, which undergo spontaneous and rapid conversion into misfolded states. These misfolded conformers, termed “seeds,” are rich in β-sheet structure that stack into one another to form higher-order oligomers and fibers. This process is commonly known as the nucleation-polymerization mechanism (1, 2, 3). Mounting evidence indicates that these oligomeric structures possess significant neurotoxicity and have the potential to recruit additional proteins, leading to the formation of proteinaceous aggregates such as Lewy bodies (LB) (4, 5, 6, 7, 8). α-Synuclein, an intrinsically disordered protein of unknown function, is recognized as the predominant component of LB. Nevertheless, the investigation into the characterization and recruitment mechanism of proteins associated with LB remains a substantial and ongoing area of research in the field (9). Biochemical studies of human post-mortem PD brain tissue reveal the accumulation of diverse ubiquitinated proteins and components of the ubiquitin-proteasome system (UPS) within the LB (10, 11, 12, 13). Proteomic investigations of LB unveil the presence of approximately 40 proteins, among them a novel deubiquitinating protein OTUB1, which is co-enriched with α-synuclein (14).

Notably, OTUB1 has been characterized as an amyloidogenic protein, and its aggregation demonstrates a positive correlation with neurotoxicity in SH-SY5Y cells. Furthermore, OTUB1 colocalizes with phosphorylated α-synuclein, a pathological hallmark of PD in mouse brains (15). OTUB1 also directly interacts with Tau, an essential protein in AD, and increases the aggregated tau loads (16). Yet, the relationship between Tau aggregation and amyloid properties of OTUB1 is unknown. Additionally, the mechanistic comprehension of the aggregation process and the underlying driving factors remain unexplored. While the association of OTUB1 in various cancers has been extensively studied, ongoing efforts to target this protein with inhibitors are in progress (17, 18, 19). However, the potential limitation of inhibitory activity due to its aggregation needs consideration. Thus, gaining a mechanistic comprehension of aggregation is imperative not just for understanding LB formation and pathology but also for optimizing inhibitor design efficacy in diseases associated with LB pathology.

OTUB1, a 271-residue OTU-family deubiquitinase, specifically cleaves K-48-linked polyubiquitin chains from its substrate (20, 21). In its apo form, devoid of substrate binding, OTUB1 maintains a closed conformation. However, substrate binding induces a small-scale structural reorganization, forming an active catalytic triad consisting of Cys-91, His-265, and Asp-267 (20). The N-terminal region, encompassing approximately 40 amino acid residues, adopts a disordered structure that plays a pivotal role in substrate specificity, localization, and regulation of diverse biological functions through protein-protein interaction (22).

Intriguingly, the N-terminal region interacts with several E2 enzymes, modulating their catalytic activity (21). Notably, it inhibits K63-linked polyubiquitination upon binding with E2 ubiquitin-conjugating enzyme Ubc13, thereby regulating cellular signaling pathways (23, 24, 25). Despite its involvement in various cellular processes like DNA break repair pathway (26), apoptosis (27), inflammation (28, 29), tumor metastasis, and various cancers (28, 30), the precise molecular function of OTUB1 remains largely unknown. It rescues p53 from degradation, induces cellular senescence, and stabilizes the Smad2/3 complex, a crucial component of the TGF-β pathway controlling diverse cellular functions (28).

Adding complexity to its properties, the globular nature of OTUB1 raises intriguing questions, particularly regarding globular, natively structured protein aggregation, a mechanism less explored in the current literature. The mechanistic understanding of OTUB1 aggregation becomes even more significant in addressing the long-standing protein folding problem. Considering its substantial physiological and pathological relevance, our study aims to unravel the molecular mechanism of OTUB1 aggregation.

Through extensive biophysical analysis and complementary atomistic molecular dynamics (MD) simulation, our research reveals that OTUB1 aggregation is facilitated by destabilization, primarily influenced by the presence of phenylalanine at position 133 and valine at position 173. These critical positions reside on distantly located surface-exposed hydrophobic patches. Additionally, our findings demonstrate that nucleation-dependent polymerization is influenced by pH fluctuation. At physiological pH, the aggregation is limited to an oligomeric state, progressing towards fibrillation as the solution pH increases. The introduction of Alanine residue at position 133 delays the overall fibrillation process, while the inclusion of a positively charged Lysine at 173 positions (V173K) completely inhibits higher-order oligomerization and fibrillation. The mechanistic insights presented here may extend to other natively folded amyloid proteins, providing valuable information in the broader context of protein folding.

## Results

### Surface-exposed hydrophobic residues at 133 and 173 drive the aggregation of OTUB1

**Figure 1:**
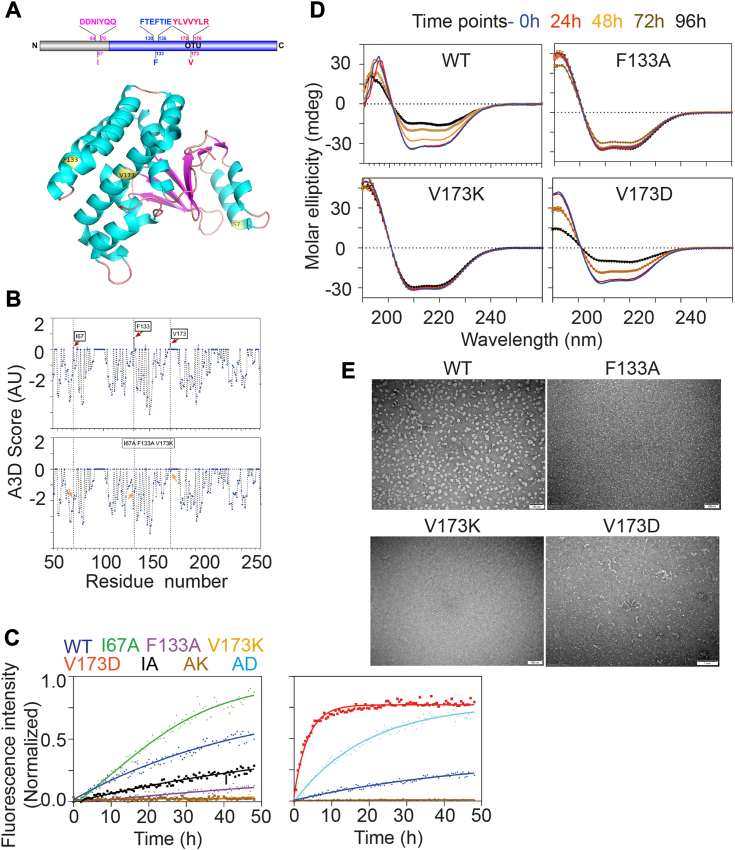
**OTUB1 aggregation is driven by two surface-exposed hydrophobic residues, F133 and V173.***A*, schematic illustration of OTUB1 structure (PDB ID-2ZFY). The locations of the mutations incorporated were highlighted in *yellow color*. The N-terminal unstructured region is shown in *gray color* and the folded region in *blue* (*upper panel*). *B*, *upper panel*-residue-wise *in silico* prediction of aggregation propensities using AGGRESCAN3D (A3D) identified I67, F133, and V173 with positive A3D scores highlighted by a *vertical dotted line*. Positive A3D scores are referred to as aggregation-prone. A3D tool utilized the 3D crystal structure of truncated OTUB1 (PDB ID-2ZFY) for the analysis. Lower panel-substituting with a lesser hydrophobic residue at I67A, F133A, and V173K/D substantially reduces the A3D score at the respective locations indicated by *arrows*. *C*, ThT binding kinetics shows the aggregation of OTUB1 (*blue*) and its different variants (n = 5). Data were fitted using non-linear sigmoidal fit in GraphPad Prism 8.0.2 (263). In the *upper panel*, mutant V173K (*dark orange*) and F133A (*purple*) show almost no ThT fluorescence indicative of reduced aggregation, and I67A (*green*) indicates slightly faster aggregation. The *lower panel* (ii) shows V173D (*red*) aggregates rapidly. For representation, extremely fast aggregating mutants V173D and F133A V173D (*teal*) were normalized separately with wild-type and plotted in the *lower panel* (ii). *D*, time-dependent CD spectroscopy measurement of secondary structural changes during the course of aggregation. Measurements were carried out at 0 h, 6 h, 12 h, 24 h, and 48 h time points of aggregation. *E*, negative staining TEM images of the final time point of aggregation of different OTUB1 mutants showing the morphology of the aggregates. Scale bar 100 nm.

A protein's folding, stability, and propensity for aggregation are intricately governed by the physicochemical properties of its constituent amino acids (31, 32). In our previous investigation, we validated that under *in vitro* conditions induced by heat, OTUB1 undergoes amyloidogenic aggregation (15). However, the specific residues or amino acid stretch responsible for conferring the amyloidogenic property of OTUB1 remains elusive. The flexibility in the N-terminal region intrigued us to presume its potential role in structural reorganization and aggregation induction. Driven by our presumption, we generated an N-terminal (45 residues) deletion construct (OTUB1ΔN) that has minimal impact on the secondary structure composition as measured by CD spectroscopy (Fig. S1, *A* and *B*). However, in the aggregation kinetics experiment using Thioflavin T dye, OTUB1ΔN displayed slightly faster aggregation kinetics than full-length OTUB1, suggesting a potential protective role against aggregation (Fig. S1*C*). Turning our attention to predicting aggregation hotspots, we employed *in silico* tools such as AGGRESCAN (33), TANGO (34, 35, 36), and Waltz (37). Notably, the recently developed AGGRESCAN3D (A3D) (38), utilizing tertiary structure information identified three residues, Ile67, Phe133, and Val173, with higher aggregation propensity scores, as shown in the A3D plot with positive peaks (Fig. 1, *A* and *B*). Interestingly, all three residues are hydrophobic, surface exposed, and situated within the helix-forming region (Fig. S1*D*). We analyzed residue-wise water solubility using Camsol (39) and observed that Ile67 resides within the soluble portion; however, both F133 and V173 are placed in the poorly soluble region (Fig. S1*E*).

Residue F133 and V173 are situated within the ubiquitin interacting interfaces (23). Interfaces involved in protein–protein interaction are notably prone to initiating aggregation, given the presence of hydrophobic patches (31, 32). Therefore, we hypothesized that reducing hydrophobicity at these locations (specifically, 67, 133, and 173) might mitigate the propensities for aggregation. In silico substitution of I67A, F133A, and V173K/V173D independently resulted in a notable decrease in the aggregation propensities as indicated by the A3D values (Fig. S1). Triple mutations specifically I67A + F133A + V173K (Fig. 1*B*) and I67A + F133A + V173D (Fig. S1, *I–K*), demonstrated a marked reduction in aggregation propensities. To validate our findings, we corroborated them with other aggregation prediction tools, Waltz and TANGO, and consistently observed that V173 yielded similar results (Fig. S1, *F*–*H*). We incorporated mutation at these specified locations and purified the variants using a recombinant expression system. Subsequently, we sought to evaluate the *in vitro* aggregation propensities of I67A, F133A, V173K, and V173D and selected double and triple mutations using Thioflavin T fluorophore binding kinetics. Our observation revealed that F133A and V173K mutations in OTUB1 effectively abrogated the aggregation. In contrast, the I67A mutation exhibited a comparatively faster aggregation than the wild-type protein. Surprisingly, the substitution of Valine to Aspartic acid at position 173 accelerated aggregation kinetics, underscoring the critical role of the local environment around the 173 positions in OTUB1 aggregation. The double mutant AD (F133A, V173D) displayed intermediate aggregation kinetics compared to wild-type and V173D mutant suggesting an independent impact of residue F133 and V173 (Fig. 1*C*). Time-dependent secondary structure analysis using CD spectroscopy and ANS binding assay supported these findings, indicating that F133A and V173K mutants resisted structural transition (α-helix to β-sheet) and hydrophobic surface exposure associated with aggregation (Figs. 1*D* and S2*A*). Electron microscopy images further revealed distinct ultrastructural differences, with V173D forming heterogeneous protofibril structure and F133A and V173K mutants resisting higher-order assembly formation (Fig. 1*E*). Additionally, fractionation of aggregates using ultracentrifugation from a kinetics study demonstrated a significant reduction in the aggregate volume, termed the Aggregation Index (AI) for F133A and V173K mutants compared to the wild-type. The V173D mutant protein is predominantly seen in the pellet fraction (Fig. S2, *D* and *E*).

**Figure 2:**
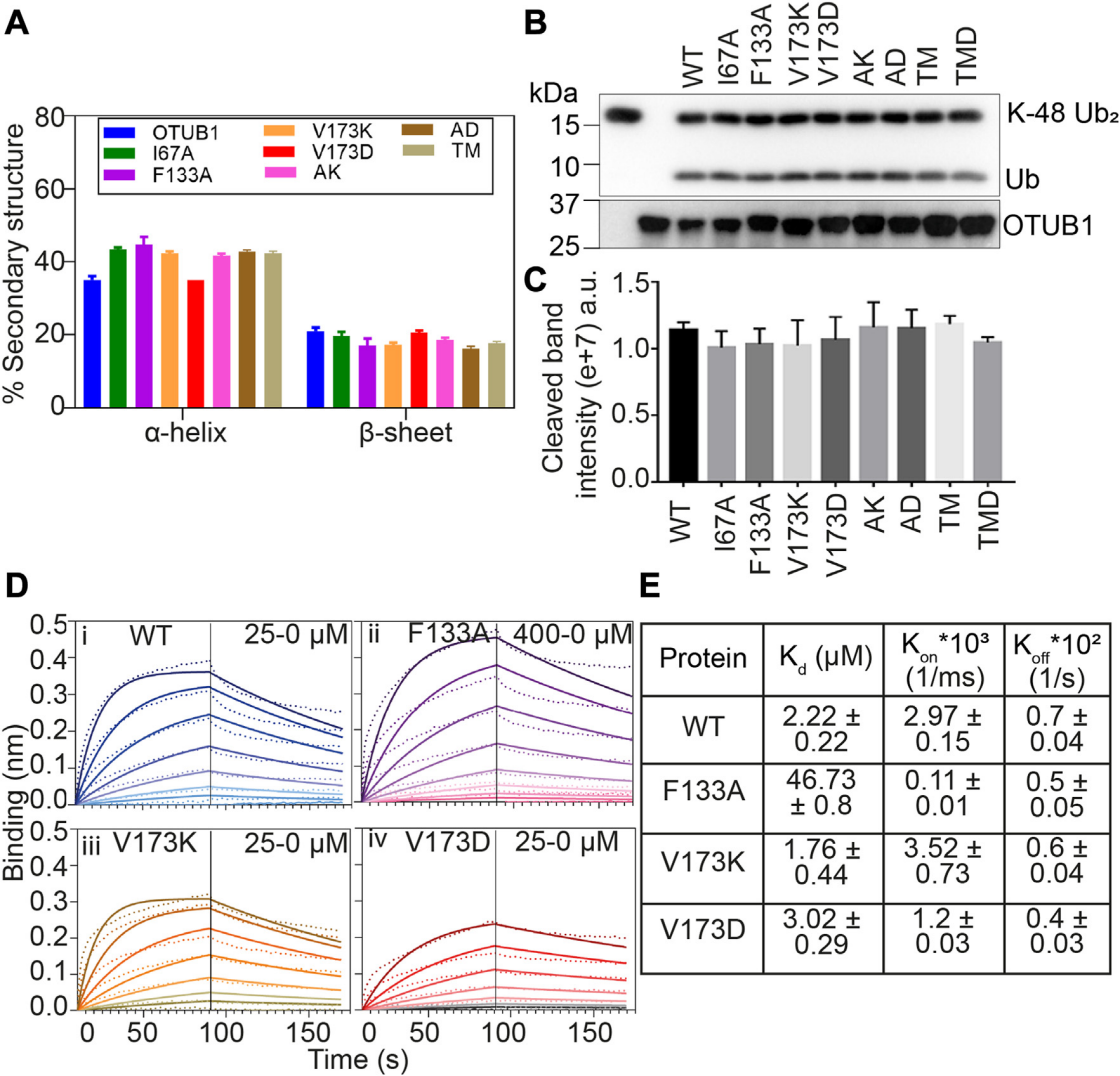
**Structural characterization of OTUB1 mutants.***A*, secondary structural changes measured by CD spectroscopy of different OTUB1 mutants. CD spectral data (n = 3) were analyzed by Dichoweb analysis, and changes in α-helix and β-sheet contents were shown in the plot. *B* and *C*, Western blot showing functional assay of OTUB1 (a deubiquitinase) mutants by diubiquitin cleavage assay. K-48 linkage-specific diubiquitin was used as the substrate, and after 1 h, the reaction was quenched, and blot was developed against an anti-ubiquitin antibody. For OTUB1, the loading control blot was probed against the anti-OTUB1 antibody. Normalized cleaved ubiquitin band intensity was plotted (n = 3). *D* and *E*, BLI was performed to show the binding of different OTUB1 variants with its interactor UBC13. UBC13 was immobilized in the AR2G sensor, varying conc. (0–400 μM) of OTUB1 and its mutants were used in the well flow. AR2G sensor without any UBC13 loaded was used as a reference and subtracted before analysis. Data were fitted using a 1:1 fitting model, and binding parameters were calculated accordingly using Data analysis HT 10.0.1.7 (Forté Bio).

Prompted by our initial observation, we delved into the potential structural and functional implications of the mutations on the protein. Employing CD spectroscopy to assess the structural properties of these mutants, we observed no noteworthy differences in the secondary structure content (Figs. 2*A* and S2*F*). Given that OTUB1 functions as a deubiquitinase enzyme (DUB), specifically targeting Lys48-linked polyubiquitin chain (2 or more ubiquitin conjugate), we assessed the catalytic activity of these mutants through a K48-linkage specific di-ubiquitin cleavage assay. Remarkably, all these mutants exhibited very similar activity as compared to the wild-type proteins (Fig. 2, *B* and *C*). These findings strongly indicate that the substitutions did not induce significant changes in the structural and catalytic properties of OTUB1. Previous structural analysis suggested that F133 and V173 are located within the proximal and distal Ubc13 binding region (23). Furthermore, they reported that the F133A mutation disrupts Ubc13 binding. To expand our insights, we performed Ubc13-binding studies with both F133A and V173K/D using Bilayer interferometry (BLI). In line with existing literature, the F133A mutation substantially abolished Ubc13 binding by approximately 21-fold (23). However, V173K or V173D mutation did not impact Ubc13 binding (Fig. 2, *D* and *E*).

### The gain in thermodynamic stability in the mutants F133A and V173K prevents aggregation

**Figure 3:**
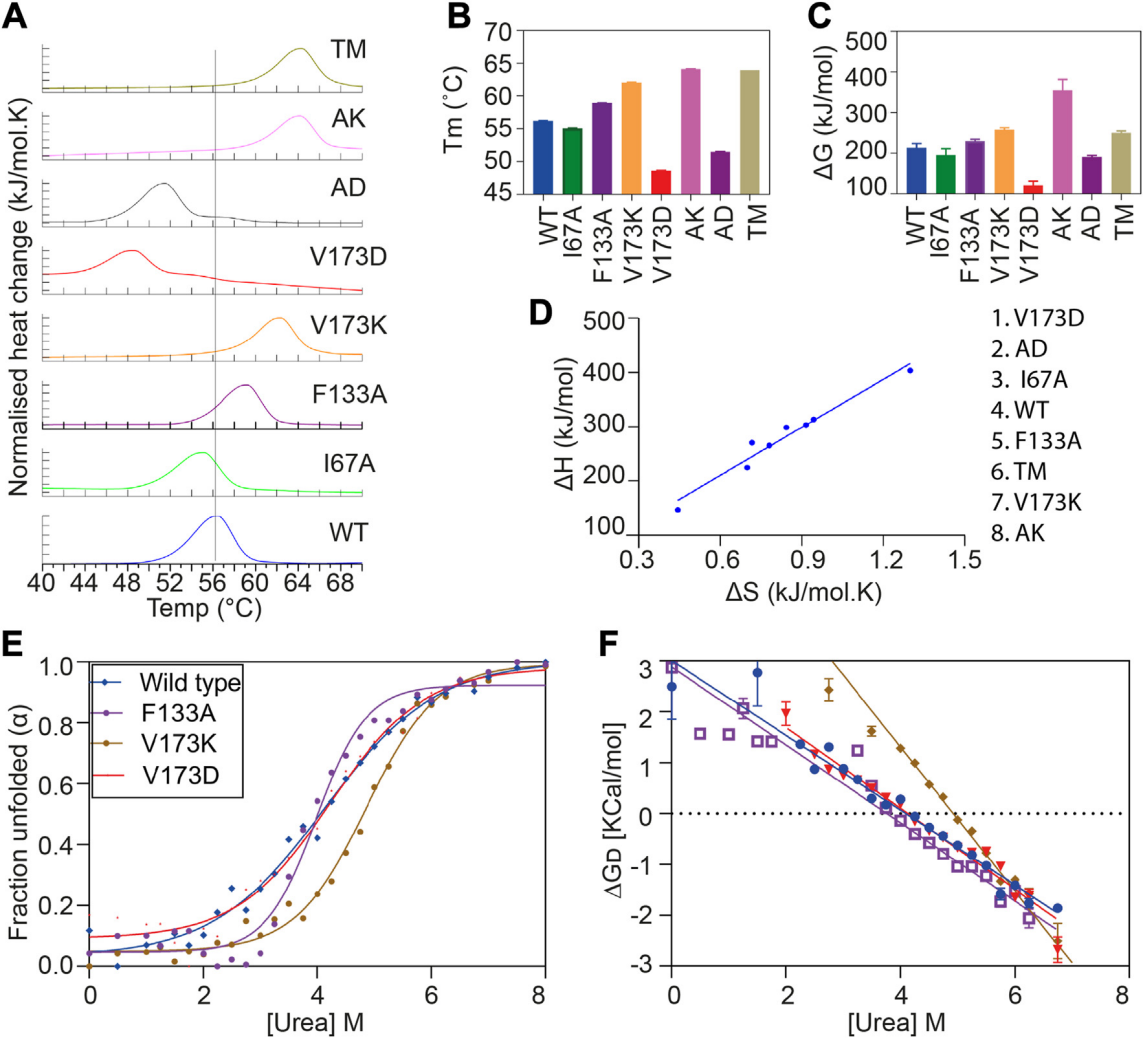
**The gain in thermodynamic stability in the mutants F133A and V173K imparts aggregation inhibition.***A*–*C*, DSC thermogram showing the thermal unfolding of different OTUB1 mutants. Normalized buffer subtracted data were fitted using two states (scaled) model, and thermodynamic parameters Tm and ΔG were calculated using Nanoanalyze software (TA system). The error bar represents the standard deviation from three independent experiments. *D*, entropy-enthalpy compensation plot generated from the enthalpy and entropy values calculated in the DSC experiment. *E*, equilibrium unfolding using denaturant urea. CD spectroscopy ellipticity was converted to the fraction unfolded, and data were fitted using a two-state fitting model. Three independent experiments were carried out, and the mean value was fitted. *F*, ΔG_D_ plot as a function of denaturant concentration. ΔG_D_ was calculated using linear extrapolation of equilibrium unfolding data.

Subsequently, we sought to unravel the mechanism by which these mutations influence the aggregation of the OTUB1 protein. The aggregation dynamics of a protein are intricately controlled by the complex interplay between its thermodynamic and kinetic properties (40, 41, 42, 43). Thus, our focus shifted to understanding the thermodynamic basis of the protein folding process. We performed thermal unfolding using differential scanning calorimetry (DSC) and CD spectroscopy (Figs. 3*A* and S3, *A*–*C*). We have fitted the protein unfolding data using a two-state fitting model. The thermogram showed a melting temperature (T_m_) of 56.2 °C for the wild-type protein. Interestingly, the Tm increased by 3 °C and 7 °C for the mutant F133A and V173K, respectively. The double mutation at these locations exhibited a further increase in the T_m_ value by 8 °C, indicating an additive effect on protein stability. Conversely, V173D displayed a reduction of 7 °C in T_m_ value, accompanied by an additional broad shoulder on the right side of the endothermic peak (Fig. 3, *A* and *B*). However, CD spectroscopy data did not show a classic two-state unfolding behavior in both the wild-type and V173D spectrum suggesting the presence of conformational heterogeneity in the native state (Fig. S3*C*). On the other hand, the I67A mutant showed a slight decrease in T_m_ (2 °C), aligning with our aggregation kinetics, suggesting a minimal role in the aggregation kinetics. We further explored other thermodynamic parameters like entropy (ΔS), enthalpy (ΔH), and Gibb’s free energy (ΔG) of unfolding. Both F133A and V173K exhibited an increase in both ΔH and ΔS but due to a larger increase in ΔH, the overall free energy of unfolding ΔG is much higher (positive) in the case of V173K followed by F133A. However, V173D showed a pronounced reduction in the ΔG (Figs. 3*C*, and S3, *A* and *B*). These data suggest that F133A and V173K mutants are more stable compared to the wild-type protein, significantly reducing aggregation. Conversely, V173D is destabilizing, possibly due to charge repulsion with a nearby negatively charged side chain, reflecting the faster aggregation of the protein. The entropy and enthalpy correlation plot (Fig. 3*D*) showed that wild-type and mutant proteins display an excellent linear correlation. The change in the entropic contribution of the system due to the mutation is compensated by a change in enthalpy, maintaining the system’s overall stability. Additionally, we performed equilibrium unfolding kinetics using a high concentration of urea monitoring unfolding by measuring absorbance at 220 nm using CD spectroscopy. We quantified the thermodynamic parameter ΔG_D_ (Gibb’s free energy change of denaturant). In wild-type OTUB1, ΔG_D_ is 3.02 kcal/mol with the midpoint of transition [urea]_50%_ at 4.1 M. V173K showed a ΔΔG_D_ of 3.96 kcal/mol (ΔG_D_ = 6.98 kcal/mol) and [urea]_50%_ (4.93 M) suggesting a thermodynamically more stable structure (Figs. 3, *E* and *F*, and Table S1). The surface exposure during unfolding, represented by a proportionality factor “m” calculated from the slope of the linear energy plot, showed a lesser value of m (−1.416 kcal/mol. M) in V173K compared to the wild-type (m of −0.736 kcal/mol. M), indicating a more compact structure that reduces the accessibility of the folded core to urea (Table S1). F133A showed a very similar ΔG_D_ and [urea]_50%_ as wild type, but the sharp transition from folded to unfolded state in the sigmoid curve suggests better cooperativity for unfolding. It provides marginal thermodynamic stability, which might be enough to reduce aggregation as compared to wild-type and V173D mutants (Table S1). In summary, our data confirm that the V173K mutant increases the overall compactness due to the close packing of the side chain, which otherwise would have been destabilizing when exposed to the bulk water.

### The structural compactness of V173K and F133A mutants protects them from aberrant oxidative damage

**Figure 4:**
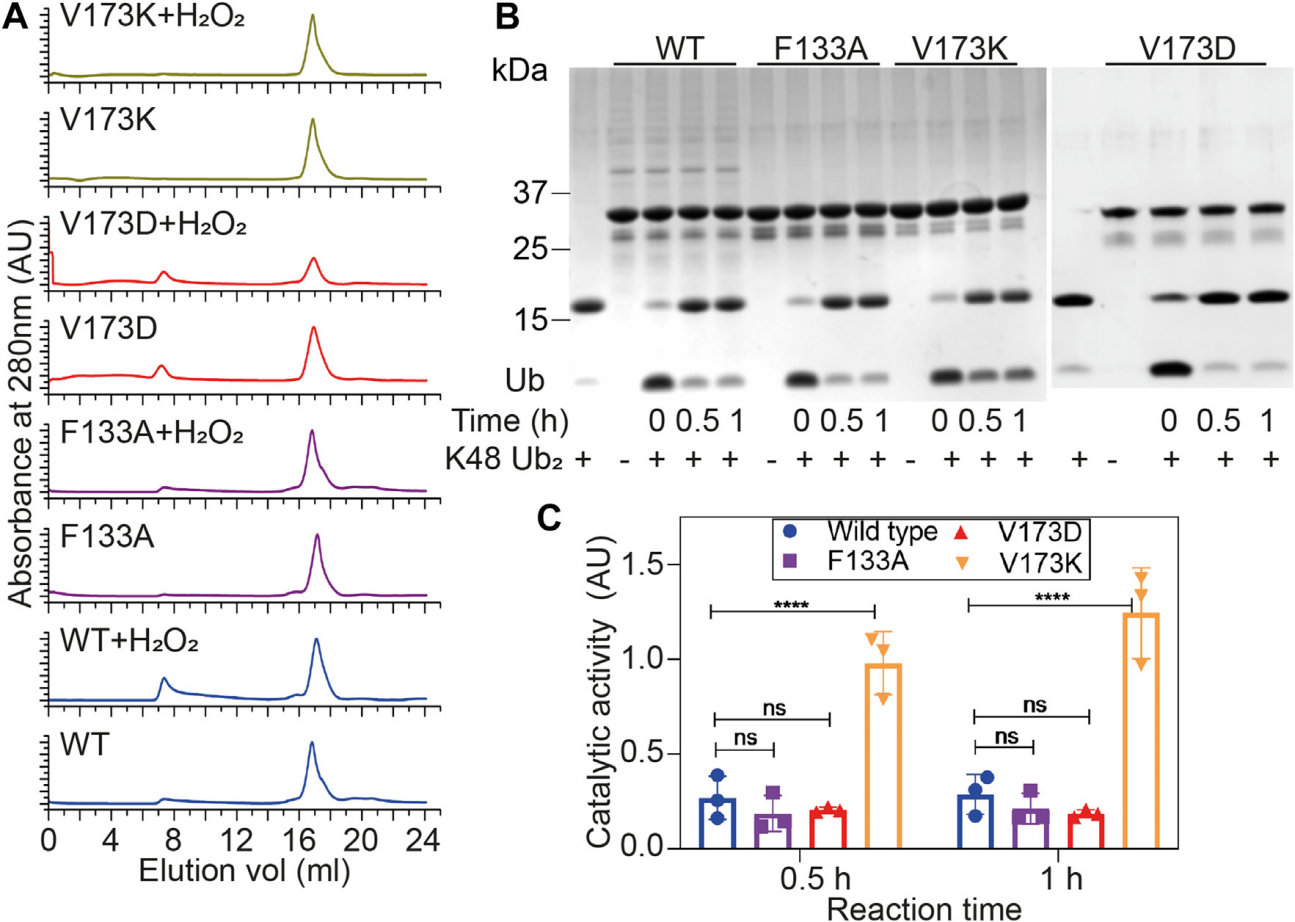
**Structural compaction in V173K and F133A protects from aberrant oxidative damage.***A*, analytical gel filtration chromatogram after treatment of wild-type protein and its mutants with H_2_O_2_ (1:20). Aggregated and misfolded components due to oxidation appeared in the void volume (8 ml) and native-like components eluted in 16 ml (elution volume). *B* and *C*, diubiquitin chain (K48-linkage specific) cleavage assay H_2_O_2-_treated proteins and catalytic activity was monitored from SDS PAGE followed by Coomassie staining. Three independent experiments were conducted, catalytic activity was calculated from the Ub/Ub2 ratio, and two-way ANOVA (Tukey’s multiple comparisons) was performed to determine the statistical significance. (∗∗∗∗ corresponds to *p*-value <0.0001, and ns corresponds to *p*-value >0.64).

High concentration of oxidizing agents, such as hydrogen peroxide, causes irreversible damage and functional impairment of proteins, primarily through the modification of Cysteine to sulfinic acid (addition of two oxygens) and sulfonic acid (addition of three oxygens) (44, 45). Accumulating evidence suggests that the proteins associated with AD and PD are highly susceptible to oxidative damage (46, 47, 48). Aberrant oxidation induces structural misfolding (collapse) and initiates protein aggregation (47). Additionally, OTUB1 aggregation is sensitive to nitrosative stress conditions (49). Given the amyloid property of OTUB1 and the presence of four Cysteine residues (C-23, C-91, C-204, and C-212), including catalytic C-91, we speculated on its susceptibility to oxidative damage. To assess this, we treated wild-type protein and its mutants with a 40-fold molar excess of hydrogen peroxide and performed gel filtration chromatography using Superose 6 10/300 Gl column to separate misfolded components. Following peroxide treatment, a substantial amount of protein appeared in the void volume, indicating extensive oxidative damage. Notably, no visible void component was observed in the case of V173K, F133A, and combined double mutant F133A V173K (AK). V173D, on the other hand, concordant with its reduced thermodynamic stability, undergoes extensive oxidative damage (Fig. 4*A*). Cysteine residues with low pKa values, particularly the catalytic Cysteine, are more vulnerable to oxidation and become functionally inactive (44). The extensively oxidized aggregated protein experiences structural collapse and is anticipated to lose catalytic activity. Our focus was on assessing the catalytic activity of moderately oxidized species that will remain in the solution. Thus, we separated the insoluble aggregated component after peroxide oxidation by high-speed centrifugation and checked the catalytic activity of the supernatant fraction using the K48-linkage specific di-ubiquitin cleavage assay. We observed that wild-type, F133A, and V173D exhibited significantly reduced catalytic activity, evident from the cleaved mono-ubiquitin band. No improvement in catalytic activity was observed even after an extended incubation time of up to 2 h. In contrast, V173K demonstrated improved catalytic activity (approximately 4-fold) compared to the wild-type, which increased with prolonged incubation time (Fig. 4, *B* and *C*).

Collectively, this experiment illustrates that in the presence of an excess oxidizing agent, the wild-type protein undergoes structural collapse, resulting in the loss of catalytic activity. However, V173K mutation, due to its enhanced compaction, is less accessible to hydrogen peroxide, maintaining structural integrity. In contrast, F133A was found to be catalytically inactive despite showing better structural integrity in the gel filtration chromatography.

### Mutant V173K escapes the conformational trap during the protein refolding process

**Figure 5:**
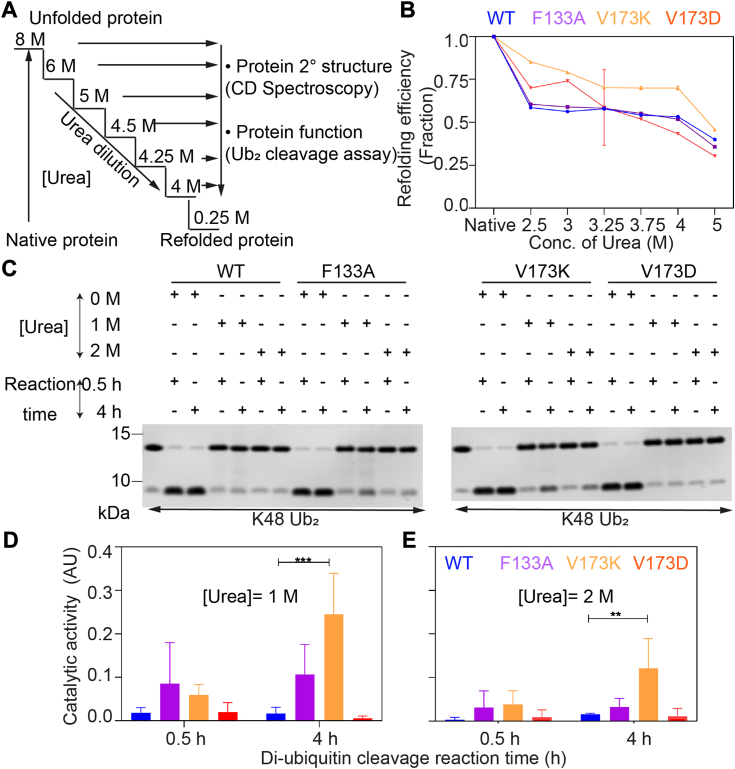
**Mutant V173K and F133A escape the kinetic trap during the protein refolding process.***A*, schematic representation of urea-mediated refolding experiment. *B*, the protein refolding efficiency plot was calculated from the refolding assay performed by CD spectroscopy, as mentioned in the schematics. Refolding efficiency was calculated, the mean was plotted with standard deviation as an error bar from three independent experiments, and statistical significance (*p*-value) was calculated using two-way ANOVA (Tukey’s multiple comparisons). *p*-value <0.0003 (∗∗∗∗), <0.0293 (∗). *C*–*E*, diubiquitin chain (K-48 linkage specific) cleavage assay of the refolded protein was checked from SDS-PAGE followed by Coomassie staining. Three independent experiments were carried out, and catalytic activity was calculated from the band intensity of the Ub/Ub_2_ ratio.

Several lines of investigation suggest that partially un/folded molten globular states that populate under physiological conditions, can drive the aggregation of different amyloid-associated proteins (50, 51, 52). We hypothesized that during the refolding process, the protein might become kinetically trapped in a misfolded molten globular state, leading to aggregation. To explore this hypothesis, we initiated the unfolding of protein using 8 M urea and allowed the protein to refold by stepwise dilution of urea (Fig. 5*A*). We monitored the refolding process by CD spectroscopy (Fig. S4) and calculated the refolding efficiency (Fig. 5). The 100% refolding efficiency means, ideally protein regains its native-like conformations with near-identical structural composition (secondary structure). Reduction in refolding efficiency provided the native condition suggests more and more populations are kinetically trapped into a partially folded conformation that can remain in the dynamic equilibrium with the misfolded conformation.

The refolding process became apparent after diluting the urea from 8.0 M to 5.0 M or below. For the wild-type protein, we observed approximately 60% refolding efficiency at 2.5 M urea. In contrast, V173K shows more than 85% refolding efficiency. Increased refolding efficiency in V173K compared to wild-type was maintained at least up to 5 M urea conc. The refolding process of F133A closely resembled that of the wild-type. However, V173D displayed a more intricate refolding pattern. Lower urea concentrations (2.5 M and 3 M) show comparatively better refolding than the wild-type (less than V173K), but above 3 M it showed the least refolding efficiency (Fig. 5*B*). The accurate refolding of the protein to the native state should reflect the catalytic activity. In the di-ubiquitin cleavage assay, refolded wild-type protein showed no significant catalytic activity at 1 M and 2.0 M urea. This suggests that the refolded ensembles are structurally different from the native protein and, therefore, become catalytically inactive. Refolded V173K, however, retains catalytic activity, albeit at a slower rate than the native protein. F133A, although it showed a similar refolding efficiency compared to the wild type, it showed a noticeable catalytic activity in the gel but was statistically insignificant. V173D, like wild-type, completely lost its catalytic activity after refolding (Fig. 5, *C*–*E*). We also investigated the catalytic activity of the refolded protein after heat-induced unfolding, which is consistent with the observation seen in urea-mediated refolding (Fig. S5). Nevertheless, this study demonstrates that wild-type and V173K follow two different refolding paths while the latter retains catalytic activity.

### The aggregation of OTUB1 is sensitive to solution pH

**Figure 6:**
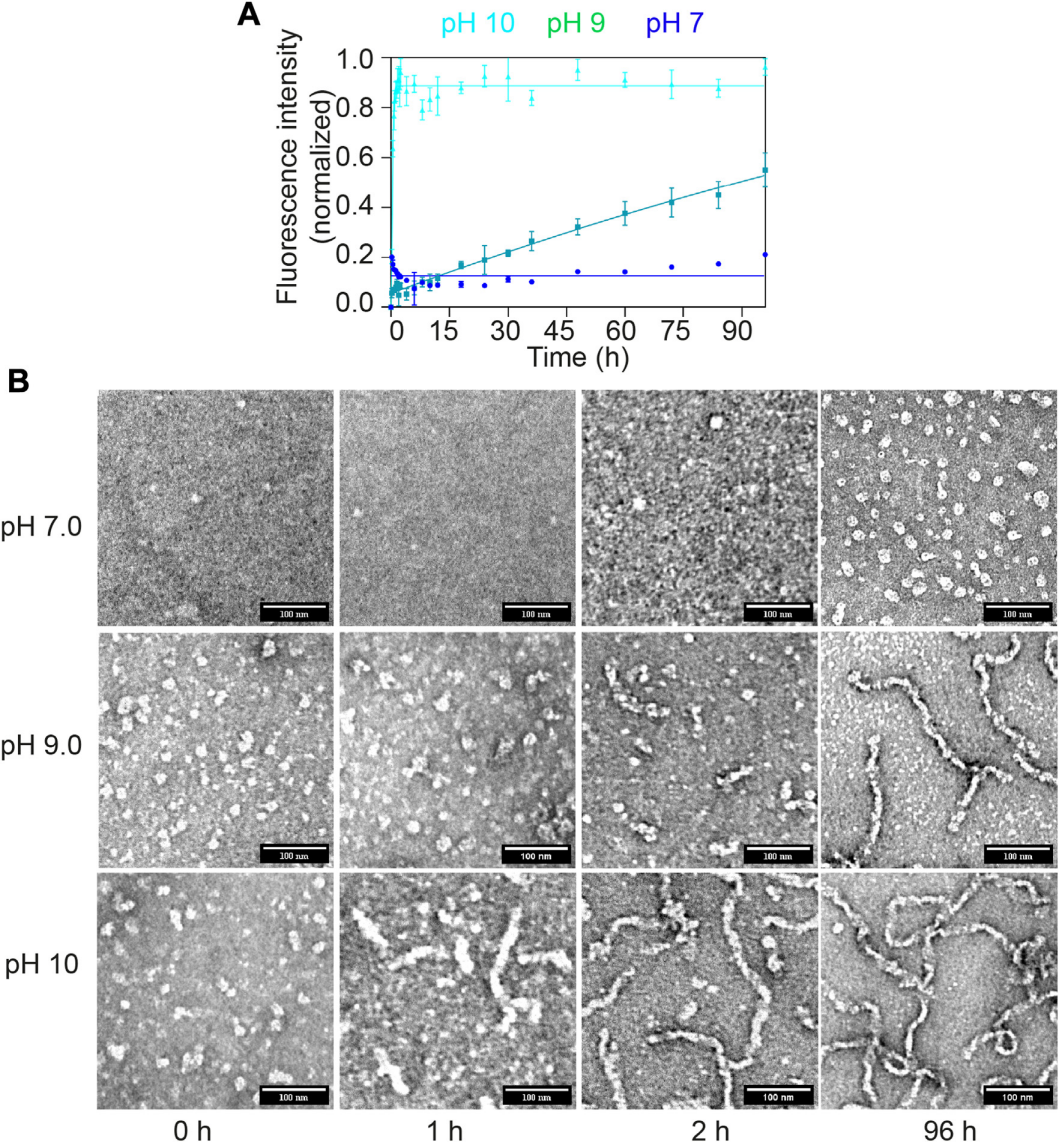
**Aggregation of OTUB1 is sensitive to solution pH.***A*, thioflavin T binding kinetics of wild-type protein aggregation at pH 7 (*blue*), 9 (*green*), and 10 (*teal*). The mean of three independent replicates was plotted, and the error bar represents the standard deviation of the mean. *B*, time-dependent changes in α-helix and β-sheet compositions were measured by CD spectroscopy of aliquots collected during aggregation kinetics. Dichroweb software was utilized to calculate the secondary structure composition. The mean of three independent replicates was plotted, and the error bar represents the standard deviation of the mean.

The pH of the solution significantly influences the protein’s stability and, subsequently aggregation. In an attempt to understand the stability of OTUB1 as a function of solution pH, we buffered protein at varying pH ranges from pH 5 to pH 10. After extensive dialysis in the desired pH, we checked the stability at 25 °C without agitation. At pH 5 to 6.5, wild-type protein was unstable and formed visible precipitations, evident from the abrupt reduction in the ellipticity value in CD spectroscopy (Fig. S6, *A* and *B*). Consequently, this pH range was excluded and the study continued at pH 7 to 10, where the protein solution remained clear without any visible precipitations. Time-dependent CD spectroscopy showed minimal changes in the secondary structure (Fig. S6*B*). To comprehend the pH-induced aggregation kinetics, we incubated proteins in the aggregating conditions at pH 7, 8, 8.5, 9, and 10. Interestingly, aggregation proportionally increases with solution pH (Figs. 6*A* and S5). The structural transition (α-helix to β-sheet) observed in CD spectroscopy facilitated rapid aggregation (Fig. S6*D*). Monitoring aggregates over time using TEM at pH 7.0, revealed that OTUB1 aggregates remained in an oligomeric form. However, at pH 9 and 10, it formed a mesh-like fibrillar structure (Fig. 6*B*). This observation suggests that a specific ionization state of OTUB1 promotes nucleation, accelerating aggregation and leading to fibrillar architecture.

### V173K and F133A resist pH-induced aggregation

**Figure 7:**
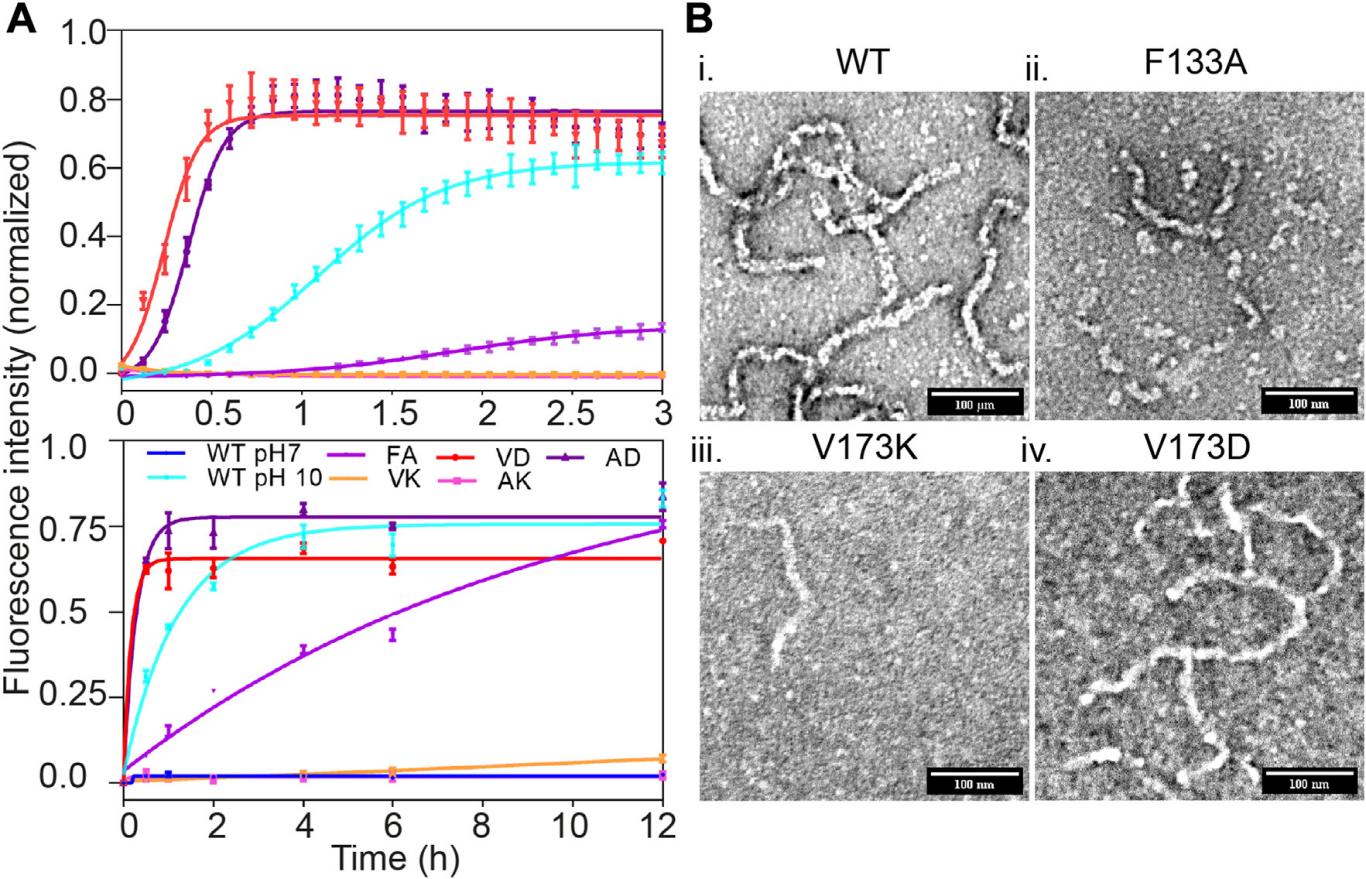
**V173K and F133A resist pH-induced aggregation.***A*, thioflavin T binding kinetics of different OTUB1 variants aggregation at pH 10. Early and late time points were shown in the upper and lower images, respectively. The mean of three to five independent replicates was plotted, and the error bar represents the standard deviation of the mean. *B*, TEM images of the aliquots collected at the endpoints of the aggregation kinetics. The brightness and contrast of the TEM snapshots were adjusted by Fiji (Image J) tool. Scale bar- 100 nm.

The thermal stability and subsequent aggregation inhibition of F133A and V173K mutants prompted an investigation of their aggregation behavior at elevated pH. Notably, the aggregation kinetics of V173K at pH 10 showed resistance to pH-induced aggregation until 12 h, while the wild-type aggregation saturated within 1.5 to 2 h. The F133A mutant at pH 10 showed some resistance but was faster than V173K. On the other hand, V173D instantly formed aggregates (Fig. 7*A*). Subsequent morphological analysis of the aggregates using TEM consistently corroborated the observed aggregation kinetics (Fig. 7*B*). In summary, stabilization conferred by structural compactness contributes to the resistance against pH fluctuations.

### Tracing the distribution of aggregates at a single molecule level

For a precise understanding of the aggregation dynamics in solution at a single molecule resolution, we employed fluorescence correlation spectroscopy (FCS). FCS, a sensitive tool for studying fluorophore fluctuations within a confocal volume due to the fluorophore’s in-and-out movement, provided insights into diffusion time (τ_D_) and hydrodynamic radius (R_h_) of protein molecule/s using Stokes-Einstein’s equation (53). The R_h_ of monomeric OTUB1 from the FCS data was found to be ∼1.95 nm (Figs. S7*A*, and Table S2), which is very close to the radius of gyration value (Rg = 1.86) calculated by using Crysol, ATSAS 3.2.1 (PDB code-2ZFY) (54).

**Figure 8:**
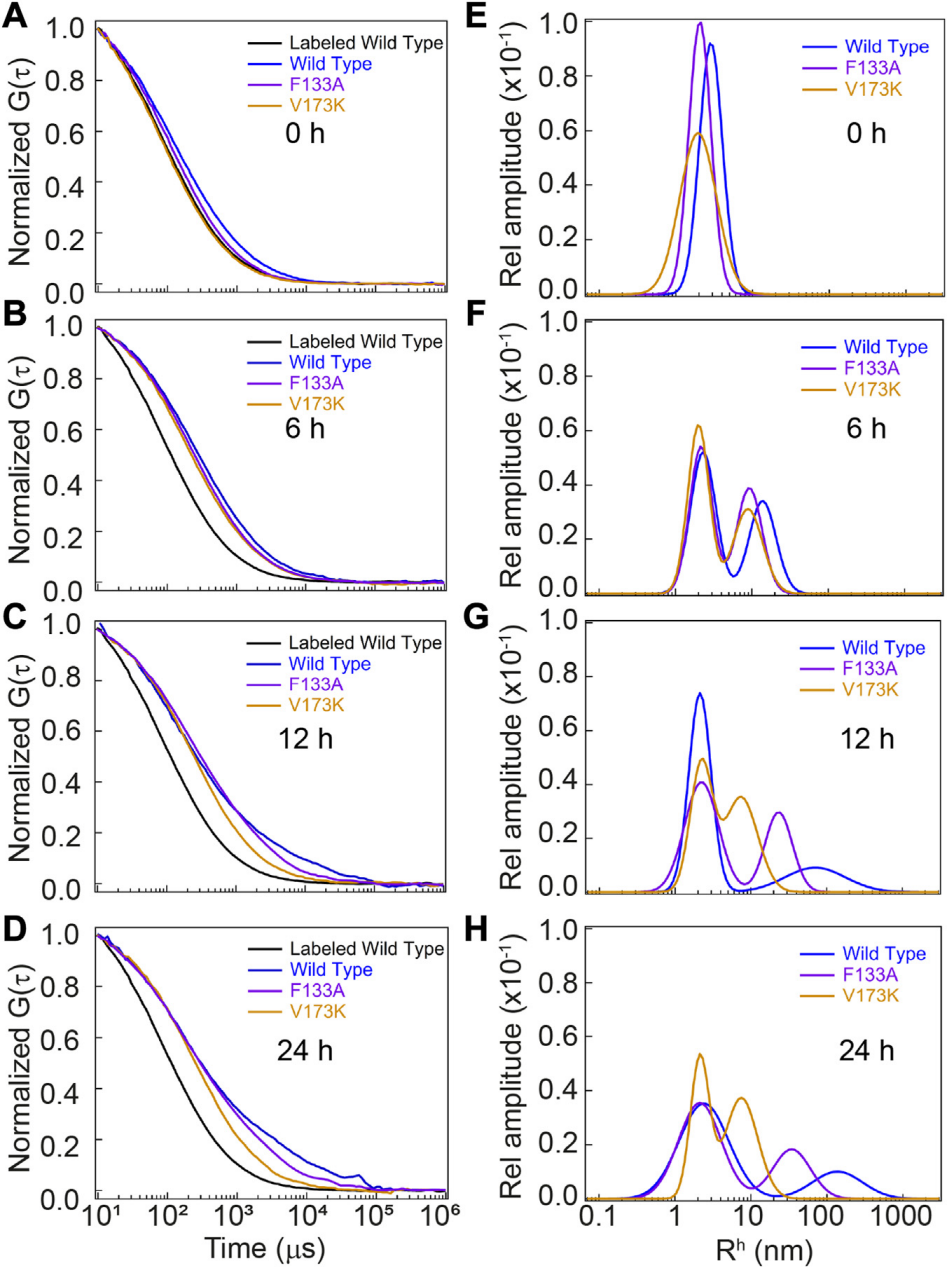
**Tracing the distribution of aggregates at a single molecule level.***A*–*D*, normalized correlation plot as a function of diffusion time (τ_D_) (log-scale) shown at different aggregation time points. Data was fitted using a single-component Gaussian distribution model (0 h) and a dual-component Gaussian distribution model (6 h, 12 h, and 24 h). *E*–*H*, Gaussian size distributions of the aggregates as calculated from the Stokes-Einstein’s equation.

At the 0-h mark, the wild-type protein shows an average R_h_ of 2.78 nm, which is a little larger than the native monomeric form, indicating the formation of extended conformers. In contrast, V173K maintained a similar R_h_ to the native state, while F133A showed a slightly increased R_h_ (2.12 nm) (Fig. 8*A* and Table S2). After 6 h, a new peak emerged in all three cases, captured by dual component GDM analysis. The wild-type displayed an average Rh of 14.02 nm for the second peak, equivalent to 6 to 7 monomer units. F133A and V173K showed an average R_h_ of 8.94 nm, equivalent to roughly 4 units (Fig. 8*B* and Table S2). As time progressed, the R_h_ value corresponding to the second peak in the wild-type rapidly increased to 132.64 nm at 24 h. In contrast, V173K showed no significant increase in the R_h_ up to 24 h, aligning with the ThT binding kinetics (Fig. 8, *C* and *D*). Although F133A exhibited an increase in R_h_, the kinetics was slower than the wild-type (Fig. 7). It is noteworthy to mention that we could not perform FCS measurements beyond 24 h of aggregation because of the frequent scattering observed by the larger oligomers in both the wild-type and F133A. The FCS measurements, complemented by DLS (Fig. S8), supported the conclusion that wild type readily undergoes oligomerization to form higher-order structures under physiological conditions. In contrast, the V173K mutant confines protein mostly to monomeric and smaller oligomeric structures, while the F133A mutant exhibits slower oligomerization kinetics.

### All-atom molecular dynamic (MD) simulation reveals the stability of mutants

**Figure 9:**
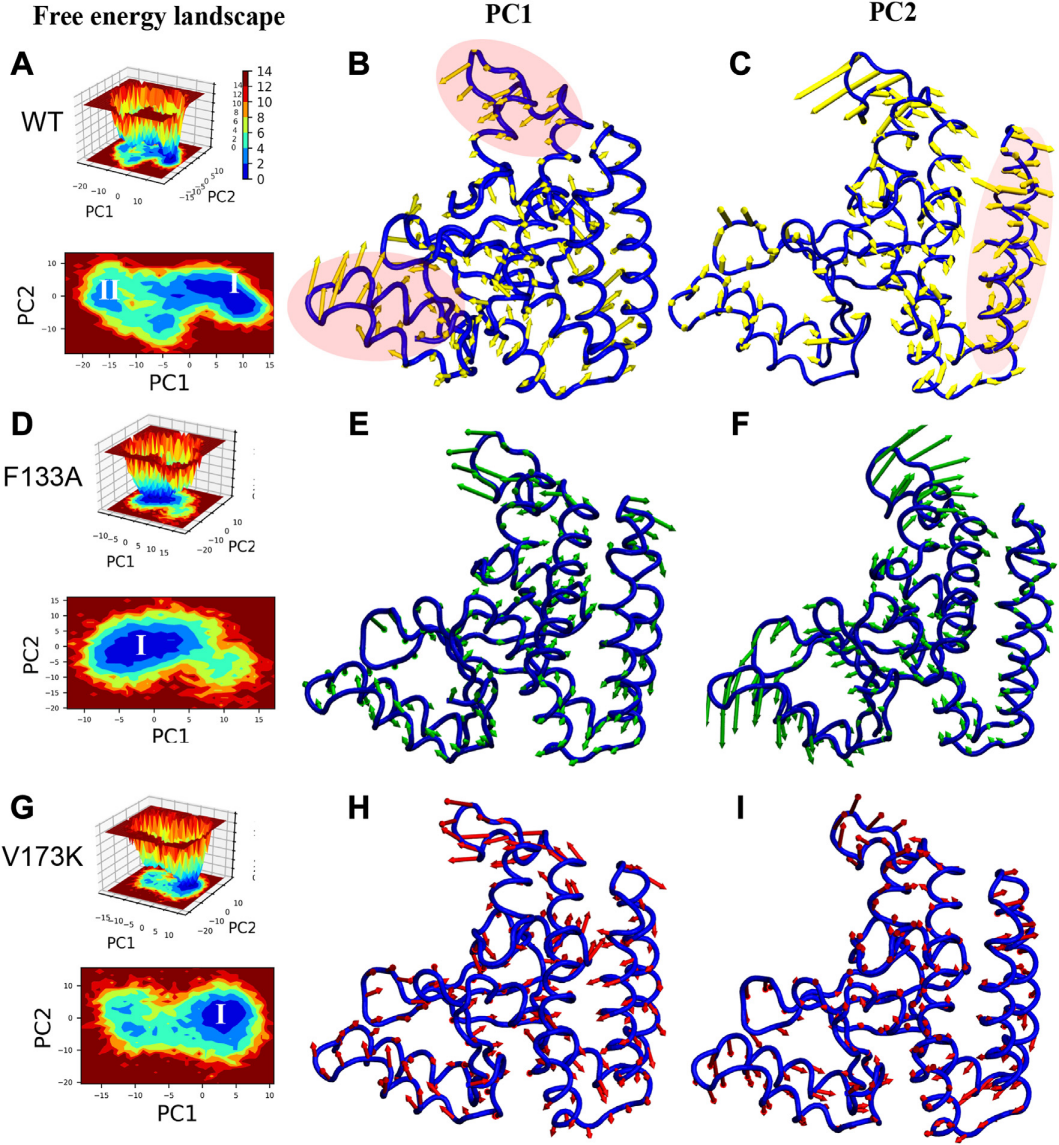
**The free energy landscape portrays mutants as the most converged system.***A*, *D*, and *G*, 2D and 3D free energy landscape (FEL) plots between PC1 (X-axis) and PC2 (Y-axis) for wild type, F133A, and V173K, respectively. The color bar represents Gibb’s free energies in the plot, ranging from the lowest energy (*blue*) to the highest energy (*red*) conformation states. The low energy minima are shown in the 2D FEL plots. *B*, *C*, *E*, *F*, *H*, and *I*, porcupine plots were generated using extreme PC1 (B, E, H) and PC2 projections (*C*, *F*, and *I*) for all the simulated systems. The direction of the arrows (in PC1 and PC2, respectively) at each Cɑ shows its direction of motion, and the length of the arrow depicts its strength. The protein is represented in tube form. Highly fluctuating regions are highlighted in the shadows.

All-atom MD simulations were executed to rationalize the difference in the stability of OTUB1 in the wild-type and mutants (F133A and V173K) utilizing PDB ID- 2ZFY. Remarkably, the root mean square deviation (RMSD) analysis unveiled bi-modal trajectories for the wild-type (apo), characterized by an initial short jump at around 30 ns, followed by a longer jump at 125 ns. In contrast, the RMSD of mutants F133A and V173K displayed a unimodal trajectory, indicating significantly reduced conformational heterogeneity in mutants. The root means square fluctuation (RMSF) analysis of Cα atoms highlighted conformational flexibility in various structural regions, notably in residues 185 to 200 and 230 to 245. However, both the F133A and the V173K mutants displayed a substantial reduction in global fluctuations, particularly in the vicinity of the incorporated mutations (Figs. 9, and S9, *A* and *C*). This diminished fluctuation in the mutants could be attributed to the gain in intramolecular hydrogen bonding interactions between residue K173 and its neighboring residues, including D169, Y170, L171, V172, Y174, and L177 (Fig. S9, *F* and *G*). Similarly, F133A showed increased stability through hydrogen bonding at T131, E132, T134, and E136 with A133 (Fig. S9, *D* and *E*). The hydrogen bonding timeline suggested that these residue pairs remain stable within 5.0 Å throughout the simulation timeline (Fig. S9, *D*–*F*). The occupancy of these residues is shown in Table S4. The gain in hydrogen bonding interaction in their vicinity might be due to the positively charged side chain/more hydrophilic side chain/longer side chain of lysine, or a combination thereof.

### Free energy landscape portrays mutants as the most converged system

To gain a more comprehensive understanding of protein structural changes, thermodynamic profiling was carried out to connect the stable lowest energy states and sub-conformational states within the protein's conformational space along principal components PC1 and PC2 (55, 56). Wild-type trajectories showed multiple energy basins at distant places, suggesting dynamic conformational changes. In contrast, V173K and F133A maintained a single, smooth, and deeper energy basin, reflecting the dynamic solidity of the conformational state and higher system stability (Fig. 9, *A*, *D*, and *G*). Moreover, the conformations extracted from PC1 and PC2, as depicted in porcupine plots, revealed the existence of global atomic fluctuations (anti-correlated motion) in the wild-type (Fig. 9). Interestingly, in the mutant systems, the overall atomic fluctuations were reduced (Fig. 9, *E*, *F*, *H*, and *I*).

A curve between free energy and biased collective variables (CV) highlighted the free energy barrier. The 1D free energy profile along PC1 and PC2 for all systems reveals that the F133A and V173K span the smallest ranges, while the wild-type covered a larger conformational space (Fig. S9*H*). Furthermore, the wild-type system diverged more when compared to mutants, as depicted by magenta and green arrows in Fig. S9*H*. This illustrates that in both mutant systems, a significant population of protein conformations is captured in a single minimum, while the wild-type traverses through different barriers.

Taken together, these observations suggest that the wild-type protein exhibits two distinct conformational ensembles separated by a thermodynamic barrier. Elevated kinetic motion facilitated the crossing of the barrier, resulting in the sustained presence of either conformation in equilibrium. Mutants V173K and F133A gain more stability by restricting the protein to a single minimum through a reduction in global residue fluctuations.

### OTUB1 co-aggregates with α-synuclein

**Figure 10:**
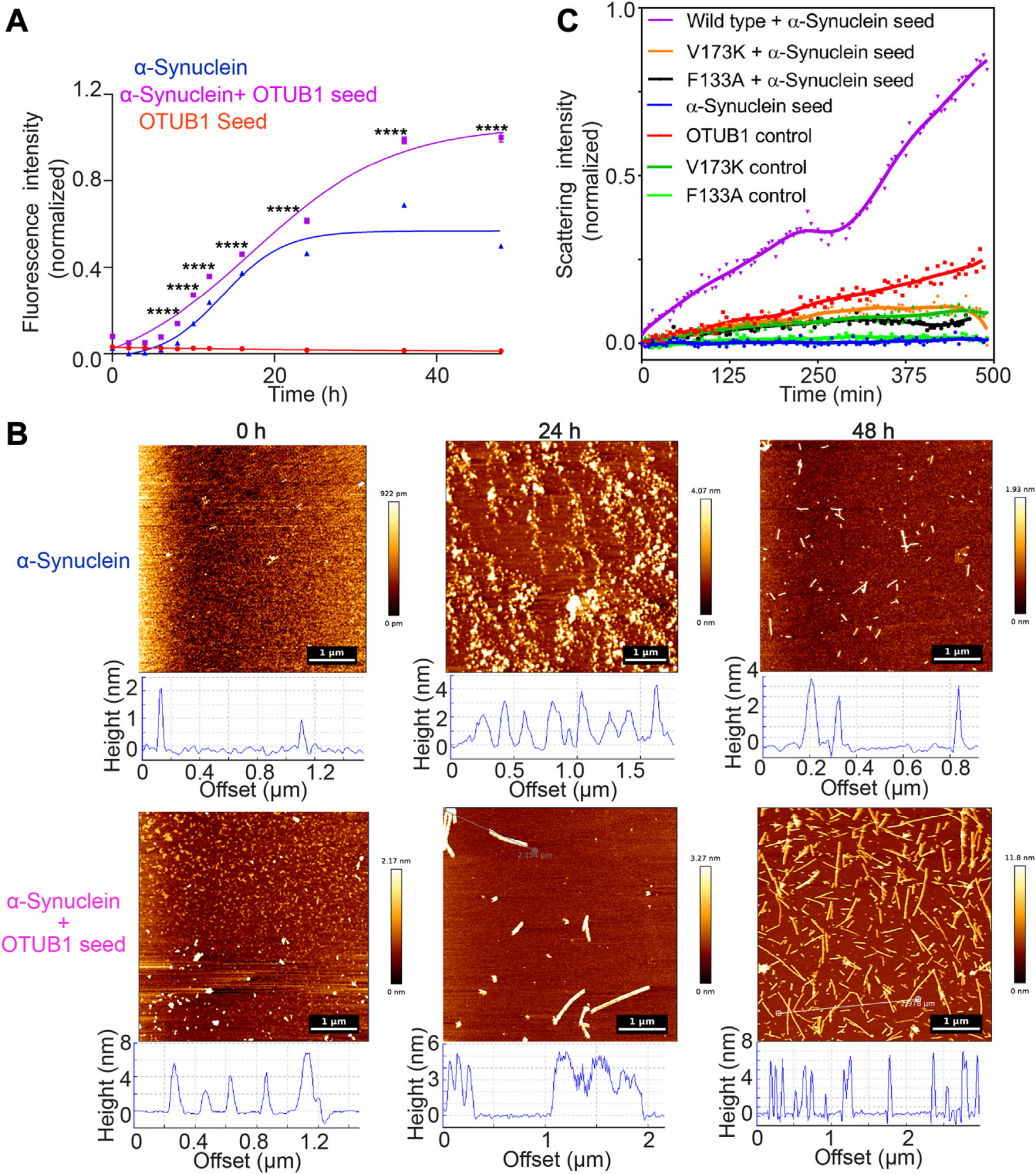
**Heterotypic aggregation: *in-vitro* coaggregation of OTUB1 and α-Synuclein.***A* and *B*, seeding effects of OTUB1 aggregates on the aggregation of α-Synuclein were studied by using ThT binding kinetics (*A*) and atomic force microscopy (*B*). OTUB1 seeds (5 μM) were added at 0 h to 300 μM of α-Synuclein and aggregation kinetics were performed using ThT dye binding assay. Aliquotes at different time intervals were collected to measure ThT fluorescence and for AFM sample preparation. The ThT data was plotted in GraphPad Prism and fitted using a non-linear fit sigmoidal model, and subjected to a two-way ANOVA (Dunnett’s multiple comparison test) to assess statistical significance. ∗∗∗∗ denotes a *p*-value of <0.0001. All the AFM samples were prepared on freshly cleaved mica surfaces, and imaging was performed in intermittent tapping mode using Bruker NanoWizard 3 (JPK instruments); data was processed using the JPK data processing tool (scale bar- 1 μm). *C*, cross-seeding assay by sonicated α-Synuclein pre-formed fibrils shows they can nucleate wild-type (*violet*) but not the mutants F133A (black) and V173K (*orange*) as observed from the light scattering assay.

Individuals affected with PD often exhibit a diverse range of phenotypes, with variations that are rarely identical among patients (57, 58, 59). Some patients even develop phenotypes that overlap with Alzheimer’s disease, schizophrenia, or epilepsy. This variability may stem from the heterogeneity in the causal factors. Recent studies have highlighted the coaggregation of proteins prone to aggregation (9, 57, 58, 59, 60, 61, 62, 63). Intrigued by this, we hypothesized, based on the presence of OTUB1 in the α-Synuclein-enriched LB (14, 15), α-synuclein might influence the aggregation of OTUB1 or vice-versa. Accordingly, we performed aggregation of α-synuclein in the presence of OTUB1 aggregating seeds using ThT kinetics. Surprisingly, OTUB1 seeds were capable of nucleating α-synuclein, leading to a reduction of the lag time of aggregation by approximately 2 h, along with a noticeable increase in the amplitude of fluorescence intensity in the saturated phase (Fig. 10*A*). Morphological analysis of these aggregates using AFM revealed that in the presence of OTUB1 seeds, α-synuclein undergoes fibrillation with a visibly accelerated elongation rate (Fig. 10*B*). While some fibrils showed bending and thickening (Fig. S10), single fibril analysis with immunogold labeling may offer further insights into seeding compatibility or co-assembly.

Additionally, we performed a reverse seeding assay using α-synuclein preformed fibrils (PFF) as seeds, assessing the aggregation of OTUB1 wild-type, F133A, and V173K through light scattering studies. Wild-type aggregation showed a pre-log phase followed by an exponential increase, while F133A and V173K showed insensitivity to α-synuclein seeds (Fig. 10*C*). While these findings are intriguing, further detailed exploration in this direction is warranted.

## Discussion

Comprehending the mechanistic foundations of LB formation and its implications in PD pathology has emerged as a key focus in contemporary research. Recent studies underscore the correlation between the co-deposition of various amyloids and the observed phenotypic variations in PD patients (61, 62, 63, 64). Consequently, investigating the presence of multiple amyloids in LB and their coaggregation has become a relevant research area. This study explores the mechanistic aggregation of OTUB1, an amyloid protein found in LB, and amyloid-β plaques in PD and AD, respectively (14, 15, 65).

Through all-atom MD simulations and comprehensive biophysical experiments, we identified F133 and V173 are critical residues governing protein stability. Substituting these hydrophobic residues increased thermodynamic and kinetic stability, reducing susceptibility to aggregation. The modulation in configurational restriction (ΔS), hydrophobic solvation (C_p_), bonding (ΔH), and surface exposure (m-value) (66) led to a more compact protein, resilient to oxidative damage and pH fluctuations, key factors in neurodegenerative diseases (44, 46, 47). OTUB1’s unfolding and refolding trajectories differ, with refolded protein unable to retain structural scaffold, losing catalytic activity, hinting off-pathway intermediates initiating aggregation.

V173K showed higher refolding efficiency, retaining catalytic activity, and suggesting conformational flexibility in the stable state. Lysine at the 173 position stabilized the protein, while aspartic acid destabilized the protein. The enigmatic nature of this charge specificity with contrasting effects intrigued us to have a closer look at the spatial distribution of the neighboring residues. Interestingly, we observed Asp169, which is just one helical turn away (5.3 Å) from Asp173 and Glu209, located in a loop region that is 7.2 Å (Cα-Cα distance) away. Additionally, an amino acid stretch (Y174, Y170, D169, S168, T167, S166, D164) whose specific ionization state and/or rotamer can be potentially repulsive. Our encouraging yet preliminary study on the α-synuclein and OTUB1 coaggregation will provide important insights into the heterotypic amyloid aggregation field.

Collectively, this study elucidates that physicochemical principles govern the stability of OTUB1, providing insights into the amyloid aggregation mechanisms. Our future work will delve deeper into the heterotypic aggregation mechanism and the role of hydrophobic patches as signals in this process.

## Experimental procedures

### Expression and purification of recombinant proteins

The expression plasmid pOPINK-OTUB1, having full-length human OTUB1 ORF, was a kind gift from Dr David Komander (Addgene No-61420). The plasmid DNA was transformed into BL21 (DE3) cells of *Escherichia coli*. Cells were grown in Luria Bertani media containing 0.1 mg/ml ampicillin at 37 °C until OD_600_ reaches 0.6, cooled immediately to 18 °C and induced with 1 mM isopropyl-1-thio-β-D-galactopyranoside (IPTG) for overnight. Cells were harvested, and the resulting pellet was stored at −80 °C until further use. Cells were resuspended in GST-lysis buffer (1× PBS, 400 mM KCl, 1 mM EDTA, a pinch each of PMSF, lysozyme, and pH 8.0) and lysed using PANDA Plus homogenizer. The lysate was centrifuged at 15,000 rpm for 60 min at 4 °C and the supernatant was collected and loaded onto a pre-equilibrated GST affinity column (Cytiva). Non-specific proteins were washed off by washing the column with three 3-column volume of GST-wash buffer (1× PBS, 400 mM KCl, 1 mM EDTA, pH 7.4, and a pinch of PMSF). The fusion protein OTUB1-GST was eluted in 2 column volume of GST-elution buffer (50 mM tris, 500 mM NaCl, 20 mM reduced glutathione, pH 7.4). GST tag was cut by overnight incubation with 1 mg of PreScission Protease (GST tagged) in dialysis buffer (1× PBS, 400 mM KCl, 1 mM dithiothreitol) at 4 °C. The GST tag was removed from the dialyzed solution by loading it onto a pre-equilibrated GST affinity column. Flowthrough containing OTUB1 protein without any tag was collected, concentrated with a 10 kD cut-off Amicon filter (GE Healthcare), and loaded onto a pre-equilibrated gel filtration column (HiLoad 16/600 Superdex 200 PG, Cytiva). The elution fractions were collected, and the purity was checked by SDS-PAGE. The pure fractions were pooled, concentrated, aliquoted, flash-frozen, and stored at −80 °C. The N-terminal 45 residues were removed from the coding region of OTUB1 and subcloned into pGEX-6P-1 (Amersham), and the resulting truncated protein, ΔOTUB1 was purified in the same way as full-length OTUB1 protein. The protein-coding frame of Ubc13 was subcloned into pGEX-6P-1, and the protein was purified in the same way as the full-length OTUB1 protein.

UBE1 (Ubiquitin E1 enzyme) in the pET28d vector was a kind gift from Dr Ranabir Das (NCBS, Bangalore, India), and Cdc34 (Ubiquitin E2 conjugating enzyme) was received from Addgene (Addgene no.-25598) and purified using the His-tag purification protocol. Briefly, *E. coli* BL21 (DE3) cells containing pDNA were grown in Luria Bertani media, induced with 1 mM IPTG overnight. Cells were harvested and resuspended in His-lysis buffer (50 mM Tris, 250 mM NaCl, 5% glycerol (v/v), PMSF, lysozyme, and pH 8.0) and lysed using PANDA Plus homogenizer. The lysate was centrifuged at 15,000 rpm for 60 min at 4 °C and the supernatant was collected and loaded onto a pre-equilibrated His trap (FF) column (Cytiva). Nonspecific proteins were washed off by washing the column with 20 column volumes of His-wash buffer (50 mM Tris, 250 mM NaCl, pH 8.0, 5% glycerol (v/v), PMSF, and 25 mM imidazole). Bound protein was eluted in 3 column volume of His-elution buffer (50 mM Tris, 500 mM NaCl, 5% glycerol (v/v), PMSF, 300 mM imidazole, and pH 8.0).

For UBE1, an additional ion exchange chromatography was performed. His trap-eluted protein was dialyzed in an ion exchange buffer (50 mM Tris, 50 mM NaCl, 5% glycerol (v/v), PMSF, and pH 8.0). The dialyzed protein was loaded onto pre-equilibrated Mono Q (5/50 Gl, Cytiva) and eluted in a 50 to 500 mM gradient of NaCl. The proteins were pooled, and concentrated, and gel filtration chromatography was performed in the same way as mentioned.

The expression constructs pET19b-Ub was a kind gift from Dr Chittaranjan Das (Purdue University). UbD77 and UbK48R in the same vector were generated by site-directed mutagenesis. Protein was expressed, harvested, and lysed, and cellular debris was cleared off in the same way as mentioned earlier. The supernatant was acidified by 0.35% (v/v) perchloric acid and dialyzed overnight in a dialysis buffer (50 mM ammonium acetate pH 4.5). The dialyzed protein was centrifuged at 15,000 rpm for 60 min. The supernatant was concentrated using 3 kD Amicon, and gel filtration chromatography was performed (HiLoad 16/600 Superdex 75 PG, Cytiva) as mentioned.

### Mutagenesis

The desired mutations in the OTUB1 were incorporated using a mutagenic overlapping primer pair. After PCR amplification, the amplified products were digested with *Dpn*I and transformed into *E-coli* DH5α competent cells. Sequencing (ATPC, RCB) confirmed the desired mutations and the mutant proteins were purified similarly to the full-length OTUB1 protein.

### Circular dichroism spectroscopy

5 μM of protein in 20 mM sodium phosphate buffer (pH 7.2) was taken in a 0.2 cm cuvette, and circular dichroism spectra were recorded in the far-UV region (190–260 nm) using a JASCO J-815 spectropolarimeter. Each spectrum was averaged over five scans (100 nm/min scan speed and 4-s data integration time). Baseline correction was performed before the acquisition of the data. Dichroweb analysis (CDSSTR method with the reference set 7) was performed to analyze the secondary structure composition of the spectrum secondary structure composition.

### Thioflavin-T (ThT) binding assay

Freshly purified OTUB1 and its various mutants were diluted to 350 μM (otherwise mentioned elsewhere) in the ThT buffer (sodium phosphate buffer pH 7.2, 150 mM NaCl, 5 mM EDTA, 1% protease inhibitor cocktail, 0.1% sodium azide, 0.35 mM Thioflavin dye). For the pH-dependent study, we used 125 μM of protein. Spectroscopic measurement was carried out in a 96-well plate reader using a Tecan Infinite F200 pro spectrofluorometer upon excitation at 448 nm and emission at 482 nm.

### 8-Anilino-1-naphthalene sulfonic acid (ANS) binding assay

10 μM of protein aliquot collected at different intervals was mixed with 20 μM of ANS reagent in 50 mM sodium phosphate buffer of pH 7.2 and 150 mM NaCl. The emission fluorescence spectrum from 400 to 600 nm was monitored upon excitation at 372 nm.

### Calculation of aggregation index

Aggregates of wild type and mutants at 96 h were ultracentrifuged at 85,000 rpm for 1 h in a Brucker ultracentrifuge. The aggregates in the pellet fraction and monomers in the supernatant fractions were collected. The amount of protein in each fraction was quantified by BCA protein estimation (Pierce BCA protein estimation kit), and the aggregation index (AI) for each protein was calculated by taking the ratio of protein present in the pellet fraction to the protein present in the supernatant fraction.

### Transmission electron microscopy (TEM)

Protein samples to be imaged were centrifuged at 10 to 15k rpm for 10 min, and the supernatant was diluted to 10 μM final concentration. 10 μl of diluted protein sample was adsorbed onto an immediately glow-discharged mesh 300 carbon-coated copper grid (TED PELLA, INC). The unbound solution was blotted off by Whatman filter paper and washed twice with Milli Q water. The adsorbed sample was negatively stained with 1% uranyl acetate for 1 min, and the excess stain was blotted off by Whatman filter paper and left to dry. Imaging was performed using a JEM1400 flash (JEOL).

### Bi-layer interferometry (BLI)

The binding of Ubc13 with OTUB1 and its variant was performed by the Octet RED Instrument (FortéBIO) in a 96-well plate format. 25 μg of Ubc13 was immobilized on amine reactive biosensors (AR2G) in 10 mM acetate buffer of pH 5.0 by using 1-ethyl-3-(3-dimethyl aminopropyl)-carbodiimide (EDC) and N-hydroxysuccinimide (NHS). Varying concentrations of OTUB1 and its different mutants (0–400 μM) in BLI buffer (50 mM sodium phosphate pH 7.2, 300 mM NaCl) were used for binding with 120 s association and 120 s dissociations at 25 °C. Data were analyzed using FortéBIO data analysis 10.0 software. Data were fitted using a 1:1 ligand binding model, and the binding constant (K_d_)was calculated accordingly.

### Differential scanning calorimetry

Thermodynamic parameters of protein folding were calculated using a differential scanning calorimeter (TA system). 2.5 mg of freshly purified protein was dialyzed against 20 mM sodium phosphate buffer (pH 7.2) at 4 °C overnight, vacuum degassed, and injected into the sample cell of the DSC instrument. The same dialysis buffer was used for buffer substruction. After injection, the protein was unfolded by heating the protein sample at a scan rate of 1 °C/min from 25 °C to 90 °C. The experiment was carried out thrice to maintain reproducibility. After buffer substruction, data were fitted using a two-states (scaled) model, and the thermodynamic parameters (Tm, ΔH, ΔG, C_p_) were calculated from the thermogram using the Nanoanalyze software provided by the TA system.

### Synthesis of K48-linked di-ubiquitin

To prepare the K48-linkage specific di-ubiquitin chain, 1000 μM each of Ub-D77 and Ub K48R, 0.4 μM UBE1, 20 μM Cdc34 in E1-activation buffer (50 mM Tris (pH 8.0), 10 mM ATP, 10 mM MgCl_2_, 0.6 mM DTT) was incubated for overnight at 25 °C. The chain reaction was terminated by adding 50 mM final concentration of pH 4.5 acetate buffer. K-48 linked di-ubiquitin was separated from the unreacted monoubiquitin on a MonoS 5/50 Gl column (Cytiva) using a gradient elution of NaCl (0–1 M). The purity of the elution fraction was checked by running SDS-PAGE. Pure fractions containing K48-linked di-ubiquitin were immediately pooled together, aliquoted, flash-frozen, and stored at −80 °C.

### *In vitro* protein oxidation assay

Wild-type OTUB1 and its different variants were oxidized with a 40-fold molar excess of hydrogen peroxide at 25 °C for 6 h. Oxidized protein was loaded onto an analytical gel filtration column, Superose 6 10/300 Gl (Cytiva, GE healthcare), to separate the misfolded components. Chromatogram data representing the void and elution volumes were plotted using Origin 9.0.

The catalytic activity of the oxidized products was performed by K-48 linked di-ubiquitin cleavage assay. For oxidation, 3 μg of protein was treated with a 40-fold molar excess of hydrogen peroxide in a time-dependent manner (0, 30, and 60 min) and reacted with 10 μg of di-ubiquitin for 30 min. The reaction was quenched immediately by adding Lamaeli buffer and running Tris-tricine gel electrophoresis.

### Urea-mediated equilibrium unfolding and refolding assay

Unfolding kinetics to measure the stability of OTUB1 and its mutants was carried out using a high concentration (12 M stock) of urea. 6 μM of each protein was incubated with varying urea concentrations (0–8 M) for overnight to bring the protein’s different conformational ensembles into equilibrating conditions. Changes in protein stability were monitored by measuring circular dichroism spectra in the far UV region (210–260 nm) using a Jasco J-815 spectropolarimeter in a 1 mm path length cuvette. Each spectrum was averaged over four scans at a scan speed of 50 nm/sec and a data integration time of 8 s. Individually, all the spectrums were buffer corrected, and the folding state of the protein was analyzed by considering ellipticity changes at 220 nm for individual proteins at each concentration of urea. The mean residue molar ellipticity (MRE) was calculated using the following equation:

$$\left[\theta \right]=\theta^{*}100^{*}M/c^{*}l^{*}n$$

where *θ* is the ellipticity in degrees, *l* is the optical path length in cm, C is the concentration in mg/ml, M is the molecular mass, and n is the number of residues in the protein.

[θ] is the mean residue molar ellipticity in deg.cm^2^.dmol^−1^. The unfolding plot was analyzed using a two-state model, and ΔG of denaturant (ΔG_D_ (H_2_O)) was calculated from the equilibrium constant (K_eq_). The ΔG_D_ (H_2_O) follows a linear relationship with the denaturant conc. From the linear extrapolation, we get apparent ΔG_D_ and the slope.

To perform a refolding assay, the protein was unfolded similarly as previously, followed by a serial dilution of urea to the desired urea concentration. To initiate the refolding process, dilution was made to keep the final protein concentration constant across the varying urea concentrations. Protein refolding was estimated by measuring the CD spectra, as mentioned earlier. We assumed complete refolding could be achieved if the CD spectrum at a particular urea concentration matched that of the native spectrum. Based on this, refolding efficiency was calculated using the equation mentioned below.

Fraction refolding efficiency (RE) = [(MRE at a particular urea concentration)/MRE of native protein].

### Fluorescence Correlation Spectroscopy (FCS)

The FCS experiment was conducted on a custom-built FCS setup employing an Olympus (IX71) inverted confocal microscope equipped with a 60× water immersion objective (NA 1.2, UPlanSApo, Olympus). The setup details have been previously described (References). Samples were excited with picosecond diode pumped solid state laser (green, 520 nm) with continuous mode (LDH-D-C-520, PicoQuant GmbH) and controlled by the multichannel picosecond diode laser driver (PDL 828 “Sepia II”, PicoQuant GmbH). A pair of lenses were utilized to expand the diameter of the laser beam up to approximately 10 mm, achieving the condition of overfilling the back aperture of the objective. The laser power at the sample position was controlled by the combination of laser driver (SymPhoTime 64 software, PicoQuant GmbH) and neutral density filters and was kept at <50 μW. The fluorescence signal emitted by the tracer particle was captured using the same objective in epi-fluorescence mode and then passed through a dichroic mirror (XF2016, Omega Optical) and an emission filter (607AF75, Omega Optical). Using a 50/50 cubic beam splitter, two fluorescence beams (corresponding to two channels) were generated and focused onto two confocal pinholes, consisting of fiber patch-chords with a diameter of 25 μm (Thorlabs). The fluorescence signal from two channels was fed into two separate single-photon avalanche diodes, SPADs (MPD, PicoQuant GmbH). MultiHarp150 TCSPC module (PicoQuant, GmbH) was used to cross-correlate the fluorescence signal of two detector channels. SymPhoTime-64 (PicoQuant GmbH) software was used for data acquisition.

Wild-type protein and its two mutants, F133A and V173K, were labeled with Atto-532 maleimide according to the manufacturer’s protocol (Sigma Aldrich, Cat no.-68499). Briefly, labeling was carried out in a 1:10 M ratio of protein to dye in FCS buffer (50 mM sodium phosphate, 150 mM NaCl, pH 7.2) at 4 °C for overnight. The reaction was stopped by adding a very high concentration of dithiothreitol. Free unbound dye was removed from the solution using Bio-Spin 6 column (Bio-Rad) according to the manufacturer’s protocol. The cysteine residues in the protein were labeled using Atto-532 maleimide dye, and the FCS measurement was carried out using 10 to 50 nM of labeled protein. The FCS data for standard rhodamine-6G (as a reference) and monomeric wild-type Atto-532 labeled proteins were fitted with single component 3D diffusion model (Equation 3), and the time-dependent FCS data for all proteins were fitted using single or dual component Gaussian Distribution Model (GDM). The R_h_ of monomeric OTUB1 from the FCS data was found to be ∼1.95 nm (Fig. S7*A*, and Table S2), which is very close to the radius of gyration value (Rg =1.86) calculated by using Crysol, ATSAS 3.2.1 (PDB code-2ZFY) (54). We did not observe any difference in FCS correlation in 10 to 50 nM labeled protein. Henceforth, all our FCS measurements were carried out using 25 nM labeled protein. Since OTUB1 aggregates in a concentration-dependent manner, we added 25 nM of labeled protein together with a high concentration of unlabeled protein (350 μM) to trace the kinetics of aggregate formation. The diffusion time essentially reflects the diffusion of the aggregates formed over time, assuming that the labeled proteins participate in the nucleation process. We checked the effect of viscosity (*η*) due to the protein concentration from at least three different dilutions and did not observe any difference in the diffusion time (Fig. S7*B*).

### FCS data analysis

In FCS experiments, the temporal fluctuations of fluorescence signal due to molecular processes within the confocal volume or in-and-out of that volume are analyzed by normalized autocorrelation function, *G*(*τ*), which is expressed as (53, 67, 68),

(1) $$G\left(\tau \right)=\frac{\left\langle \delta I\left(t\right)\delta I\left(t\;+\;\tau \right)\right\rangle }{{\left\langle I\left(t\right)\right\rangle }^{2}}$$

Where <*I*(*t*)> represents the temporally averaged total fluorescence intensity, *δI*(*t*) represents a fluctuation in fluorescence at time *t* relative to the temporal average ⟨*I*(*t*)⟩, and *δI*(*t* + *τ*) is the fluctuation at a later time (*t* + *τ*). Hence, the fluctuation and average fluorescence can be written (46) as,

(2) δI(t)=I(t)−⟨I(t)⟩

The equation which can model the correlation functions obtained in an FCS experiment under the assumption of Gaussian-shaped confocal observation volume is given by (53, 68, 69),

(3) $$G\left(\tau \right)=\frac{1}{\left\langle N\right\rangle }{\left(1\;+\;\frac{\tau }{\tau_{D}}\right)}^{-1}{\left(1\;+\;{\left(\frac{r}{l}\right)}^{2}\left(\frac{\tau }{\tau_{D}}\right)\right)}^{-1/2}$$

Where <*N*> represents average number of tracer particles in the confocal volume, *τ*_*D*_ represents diffusion time, *r* is the focal volume lateral radius, and *l* is the one-half distance of focal volume along the optical axis. Translational diffusion constant, *D,* can then be related to *τ*_*D*_ as (53, 68, 69),

(4) $$D=\frac{r^{2}}{4\tau_{D}}$$

If *D* is known, the hydrodynamic radius ($R_{h}$) of the particle can be obtained from Stokes-Einstein’s equation as (69),

(5) $$R_{h}=\frac{kT}{6\pi \eta D}$$

Where *k* denotes Boltzmann’s constant, *T* denotes temperature (in kelvin), and *η* represents the solvent's viscosity through which the tracer particle diffuses. Our home build FCS setup was calibrated with a standard dye rhodamine-6G (Rh6G) in water using its known diffusion coefficient of 4.14 × 10^−6^ cm^2^ s^−1^ (70). In the present FCS setup, the diffusion time of Rh6G in water is ∼35 μs. For this condition, the radial length (r) and the effective volume (Veff) were measured to be 235 nm and 0.56 fl, respectively. All the measurements were done at room temperature. We analyzed the time-dependent FCS data using a (single or double) Gaussian Distribution Model (GDM) to measure the size distribution of the protein aggregates. The fitting equation of GDM is given as (53, 69, 70, 71).

(6) $$G\left(\tau \right)=\sum_{i=1}^{m}a_{i}\left(\tau_{D_{i}}\right){\left(1\;+\;\frac{\tau }{\tau_{D_{i}}}\right)}^{-1}{\left(1\;+\;{\left(\frac{r}{l}\right)}^{2}\left(\frac{\tau }{\tau_{D_{i}}}\right)\right)}^{-1/2}$$

Where,

(7) $$a_{i}\left(\tau_{D_{i}}\right)=A_{i}\;\exp\left[-{\left(\frac{\ln\left(\tau_{D_{i}}\right)-\ln\left(\tau_{p}\right)}{b}\right)}^{2}\right]$$

and, *A*_*i*_ is the relative amplitudes of components, $\tau_{p}$ is the peak of the Gaussian distribution and *b* is the width of the Gaussian distribution. Figure 7 shows the distributions of hydrodynamic radius (*R*_*h*_) of proteins and its mutants at different time points, which are calculated by combination of Equations 4 and 5 from $a_{i}\left(\tau_{D_{i}}\right)$ *versus* $\tau_{D}$ distributions obtained from GDM analysis. All time points were fitted with dual components Gaussian distribution model except 0 h time point (single component Gaussian distribution model).

### Dynamic light scattering (DLS)

As mentioned earlier, 350 μM of protein (Wild-type, F133A, and V173K) was incubated in the aggregating conditions, and aliquots at different time intervals (0 h, 12 h, and 24 h) were collected. The size distribution of the aggregates of each aliquot (0 h, 12 h, and 24 h) was measured by Zetasizer Nano ZS (Malvern instrument), and Gaussian intensity distribution (%) as a function of size (d. nm) was plotted for each protein.

### Protein preparation and mutant generation

Wild-type OTUB1 protein structure (the apo form) was retrieved from the RCSB protein data bank (PDB: ID- 2ZFY, resolution 1.69 Å) (20). Using this structure, mutants F133A and V173K were generated using Maestro (Schrödinger Release 2022-1: Maestro, Schrödinger, LLC, New York, NY, 2020) (72). All systems were prepared using the Protein Preparation Wizard module of Maestro (73). In the preparation process, the hydrogen and bond orders were added using PRIME. The H-bond optimization and restrained minimization were also done for the systems using the OPLS3 force field model (72).

### Atomistic MD simulation

MD simulations were conducted to observe the dynamics of wild-type and mutant systems. All the systems (wild-type, F133A, and V173K) were subjected to 300 ns simulation. All details related to simulated systems are described in Table S3. The atomistic simulations were conducted using AMBER22 package using the LEaP module. To prepare the systems, we used the AMBERff14SB force field. The counterions were added to neutralize the systems. To solvate the systems, the TIP3P water model was used with a cubic box of 12.0 Å from the periphery of proteins. To remove any bad contacts and stearic clashes, the systems were minimized in three steps; first, for 2000 steps, out of which 1000 steps were used by the steepest descent method, followed by 1000 steps using the conjugate gradient. Further, position restraints of 10.0 and 2.0 kcal/mol/Å^2^, respectively, were applied to the whole protein system to relax the solvent molecules. The final stage of minimization was carried out unrestrained. The systems were gradually heated for 20 ps from 0 to 300 K. The systems were equilibrated for 500 ps at 300 K and 1 atm pressure. Subsequently, the molecular systems were simulated for 300 ns with a time step of 2.0 ps using different seed values and recording the coordinates at every 20 ps to obtain a total of 15,000 frames (55, 74).

### Principal component analysis (PCA) and free energy landscape (FEL)

To identify essential motion and conformational ensemble, we further intended to use PCA and FEL in apo and mutant systems (55, 56). The PCA calculations were performed by AMBER tools using the CPPTRAJ program over Cα atoms for all the simulated systems. Firstly, the covariance matrix C is calculated, which is defined by the equation mentioned below-

(8) $$\text{Cij}=\left\langle \left(\text{Xi}-\left\langle \text{Xi}\right\rangle \right)\left(\text{Xj}-\left\langle \text{Xj}\right\rangle \right)\right\rangle \left(i,j=1,2,3_{\cdots },3N\right)$$

where Xi and Xj are the Cartesian coordinates of the i^th^ and j^th^ Cα atom, N is the number of Cα atoms considered, and ⟨Xi⟩ and ⟨Xj⟩ represent the time-averaged over all the configurations obtained in the MD simulation. Then, the PCs are obtained by diagonalization and solving the eigenvalue and eigenvectors for the covariance matrix C. The porcupine graphs were generated using the “nmode” files to further quantify the atomistic fluctuations. Consequently, the free energy landscape was performed to explore the conformational trajectory of protein based on the PCA using the ‘g_sham’ module (56, 75). The free energy, ΔG(X), is calculated by the equation-

(9) Gα=−kTlnP(qα)/Pmax(q)

Where k is the Boltzmann constant, T is the temperature of simulation, P (q α) estimates the probability density function obtained from a histogram of the MD data, and P max(q) is the probability of the most populated state.

### Cross-seeding assay

Preparation of α-synuclein pre-formed fibrils (PFF)- α-synuclein was purified, prepared low molecular weight (LMW) species and generated fibrils similarly as described (47). OTUB1 aggregates were generated as described (15). Preformed fibrils were generated by sonicating the fibrils at 20% amplitude for 1 min 5 μM of OTUB1 seeds were used to aggregate 300 μM of LMW α-synuclein and followed the same aggregating condition as described (15). AFM samples were prepared and imaging was performed as described (15).

For light scattering assay, 100 μM of monomeric OTUB1 was incubated with 5 μM of α-Synuclein PFF at 37 °C in static condition. Scattering was visualized in Hitachi F-7000 spectrofluorometer, in time scan mode, using the same excitation and emission wavelength (500 nm, 1 nm slit), at 0.5 s integration time. Data were plotted in GraphPad Prism version 8.0.2 (263) without any fitting.

### Data availability

The data reported in this manuscript are available from the corresponding author on request. The PDB database (https://www.rcsb.org/) was utilized to obtain the crystal structures. The structures were prepared from a licensed version of the Schrodinger software suite. For MD simulations, we used the licensed version of AMBER16. The graphs were prepared using XMGRACE, and visual analysis was performed using VMD (76) software.

## Supporting information

This article contains supporting information.

## Conflict of interest

The authors declare that they have no known competing financial interests or personal relationships that could have appeared to influence the work reported in this paper.
