# Supplementary material for: Hotspot site microenvironment in the deubiquitinase OTUB1 drives its stability and aggregation
Source: J Biol Chem. 2024 Apr 23;300(6):107315. doi: 10.1016/j.jbc.2024.107315 (PMC11154711; doi:10.1016/j.jbc.2024.107315)
Supplement: Supporting Figures S1–S10 and Tables S1–S4 [file mmc1.docx]

**Supporting information**

**Hotspot site microenvironment in the deubiquitinase OTUB1 drives its stability and aggregation.**

Sushanta Majumder^1^, Mitul Srivastava^2^, Parvez Alam^3^, Sandhini Saha^1^, Raniki Kumari^1^, Ajay Kumar Chand^3^, Shailendra Asthana^2^, Sobhan Sen^3^ and Tushar Kanti Maiti^1^*

^1^Functional Proteomics Laboratory, Regional Centre for Biotechnology, NCR Biotech Science Cluster, Faridabad, 121001, India.

^2^Translational Health Science and Technology Institute, NCR Biotech Science Cluster, Faridabad 121001, India.

^3^Spectroscopy Laboratory, School of Physical Sciences, Jawaharlal Nehru University, New Delhi 110067, India.

^*^To whom correspondence should be addressed. E-mail: [tkmaiti@rcb.res.in](mailto:tkmaiti@rcb.res.in)


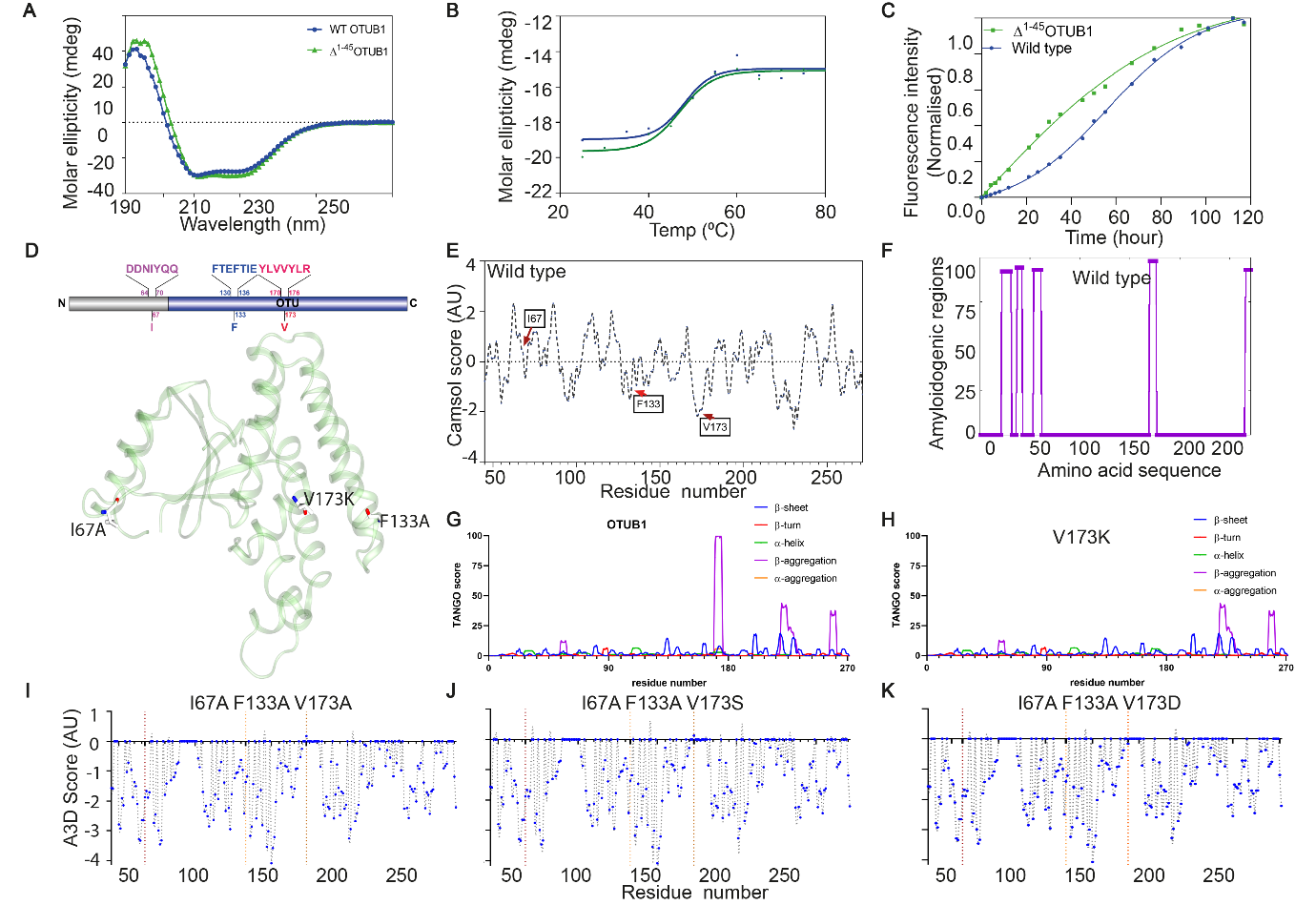


**Figure S1**: **Identification of hotspot residue associated with OTUB1 aggregation.**

**A-C**: Characterization and aggregation of full length and Δ^1-45^ OTUB1- CD spectrum (A), thermal denaturation (B), and Thioflavin T binding kinetics (C).

**D**: Residues identified with high aggregation propensities in the A3D prediction tool was shown in the crystal structure (PDB code- 2ZFY).

**E**: Residue-wise water solubility analysis of OTUB1 using Camsol

**G-H**: Aggregation prediction using other available tools like Waltz (F), and TANGO (G) shows residue 170-175 possesses consistently high aggregation propensity.

**I-K:** In silico mutational analysis in TANGO (H) and A3D (I-K) shows substituting by hydrophilic or lesser hydrophobic amino acid reduces the aggregation propensities.


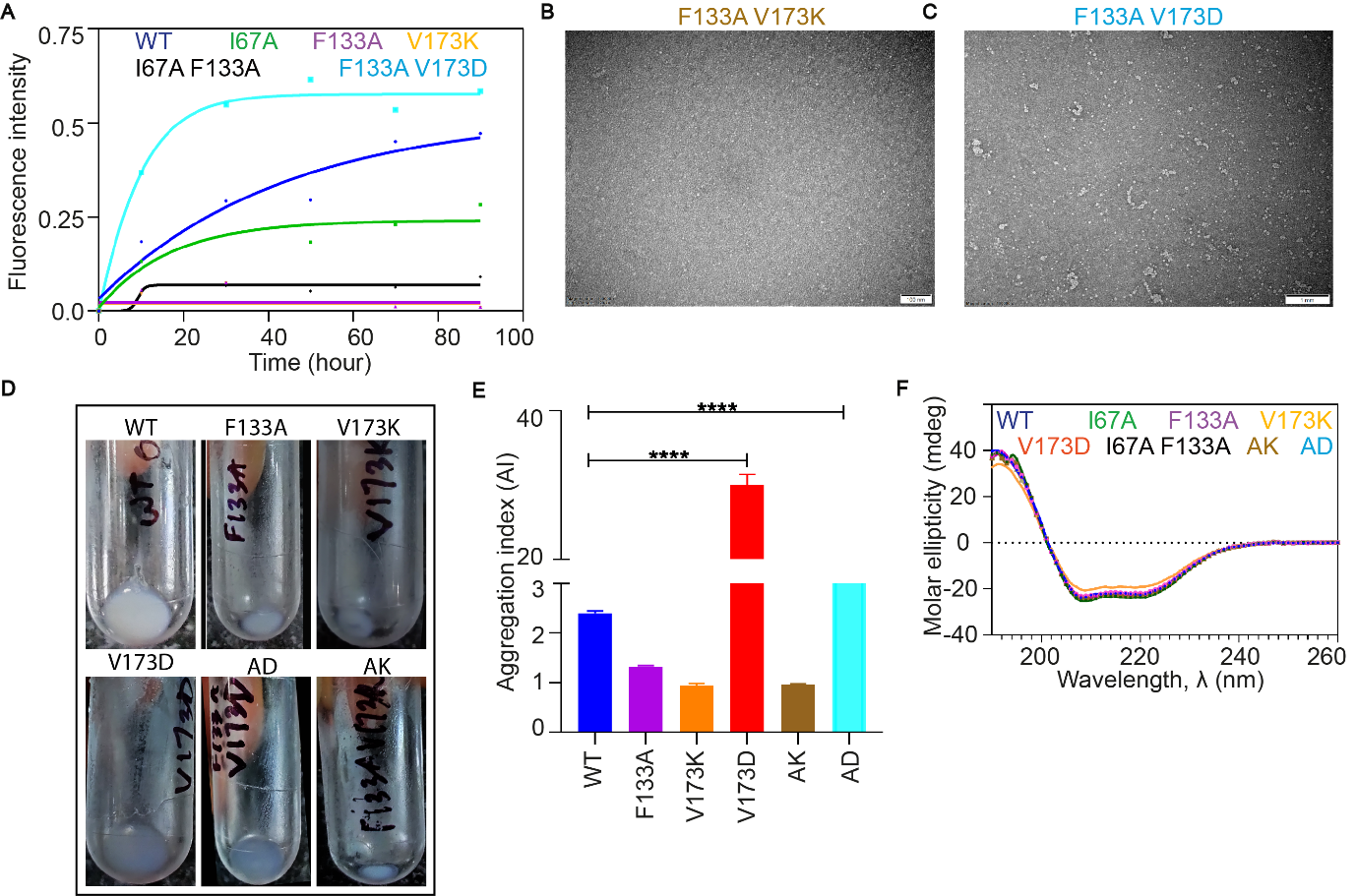


**Figure S2**: **Aggregation of different OTUB1 variants.**

**A**: ANS binding assay showing hydrophobic surface exposure during the aggregation.

**B-C**: TEM images of double mutants F133A V173K (S2B) and F133A V173D (S2C), scale bar represents 100 nm.

**D-E**: Aggregates formed at the endpoint of the kinetics were ultracentrifuged at 75,000 rpm (S2D). The amount of protein present in the pellet and supernatant fraction was quantified and the aggregation index (AI) was plotted (S2E). The mean value of the data from three independent samples was plotted and error bars represented standard deviation. Ordinary one-way ANOVA (Dunnett’s multiple comparisons test) was performed to calculate statistical significance. (**** corresponds to p-value <0.0001).

**F**: CD spectrum of wild-type and its different mutants showing minimal perturbation in secondary structure content. Spectrum from three independent measurements were averaged and the mean value was plotted for each protein.


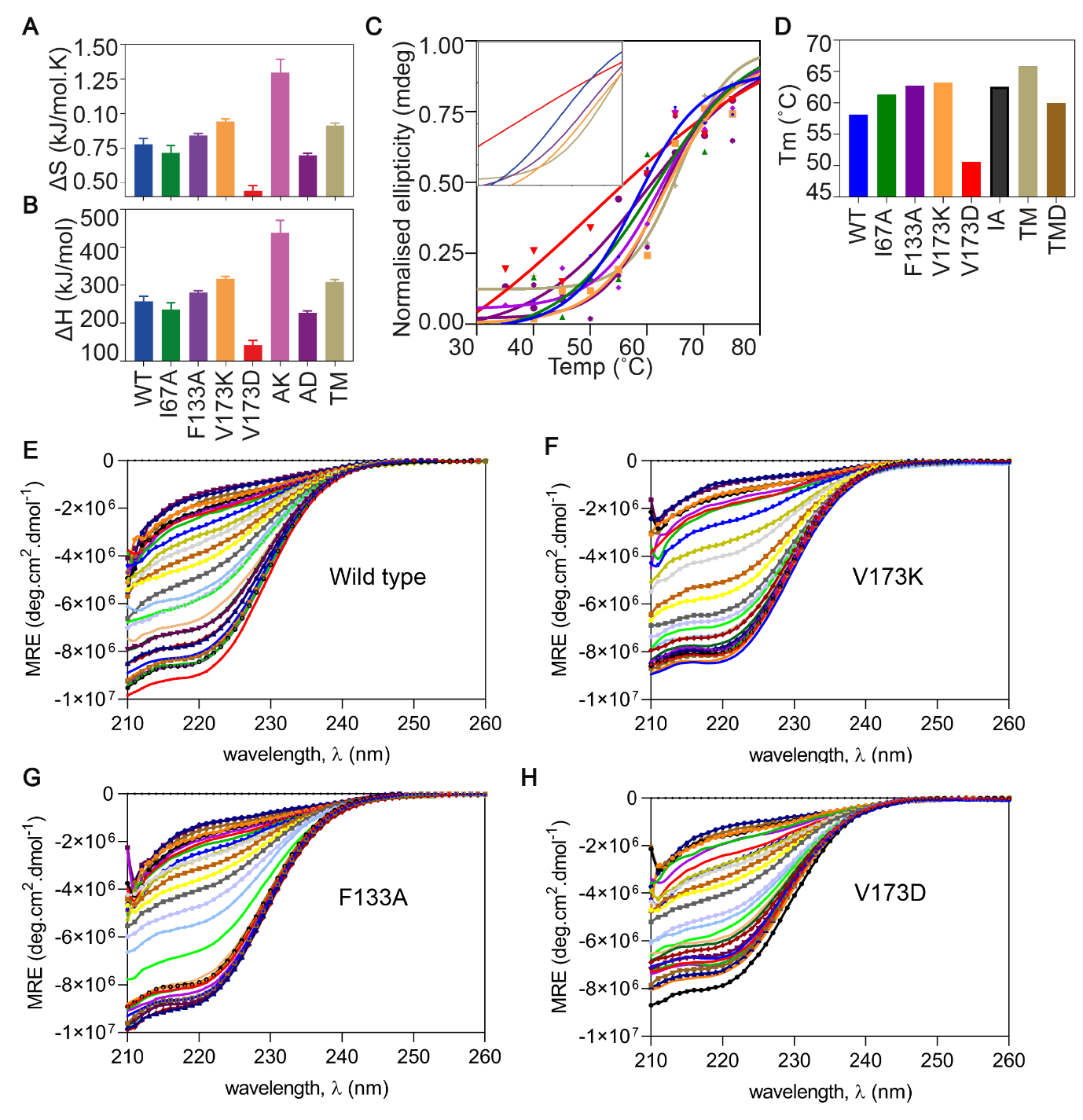


**Figure S3:** **The thermodynamic properties of OTUB1 mutants.**

**A-B**: Thermodynamic parameters change in entropy and enthalpy as calculated from the DSC thermogram shown in Figure 3 by using Nanoanalyze software (TA system). The error bar represents the standard deviation from three independent experiments.

**C-D**: Thermal melting using CD spectroscopy. The ellipticity value at 220 nm was recorded and the fraction unfolded was plotted against the temperature ramp from 30-80 °C. Melting temperature (T_m_) was plotted for each mutant (D).

**E-H**: CD spectrum showing equilibrium unfolding at varying urea concentration (0-8 M). The mean value from three independent samples was plotted.


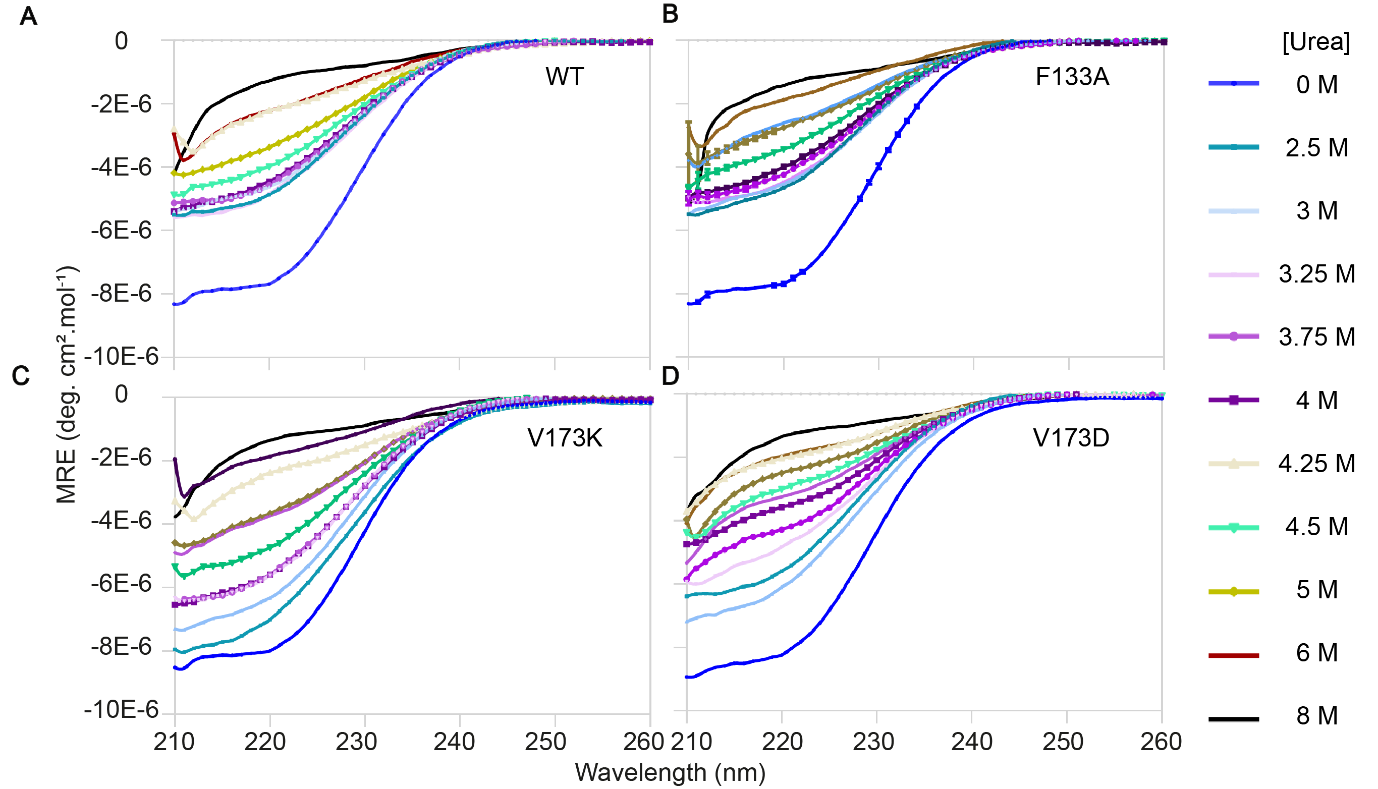


**Figure S4:** **Protein equilibrium refolding.**

**A-D:** CD refolding spectrum at varying urea concentration (8-2.5 M). Respective protein was unfolded using 8 M urea and diluted in a refolding buffer with decreasing urea concentration. CD spectroscopy measurements were performed and MRE_220 nm_ was considered for quantifying refolding efficiency. The mean value from three independent samples was plotted.


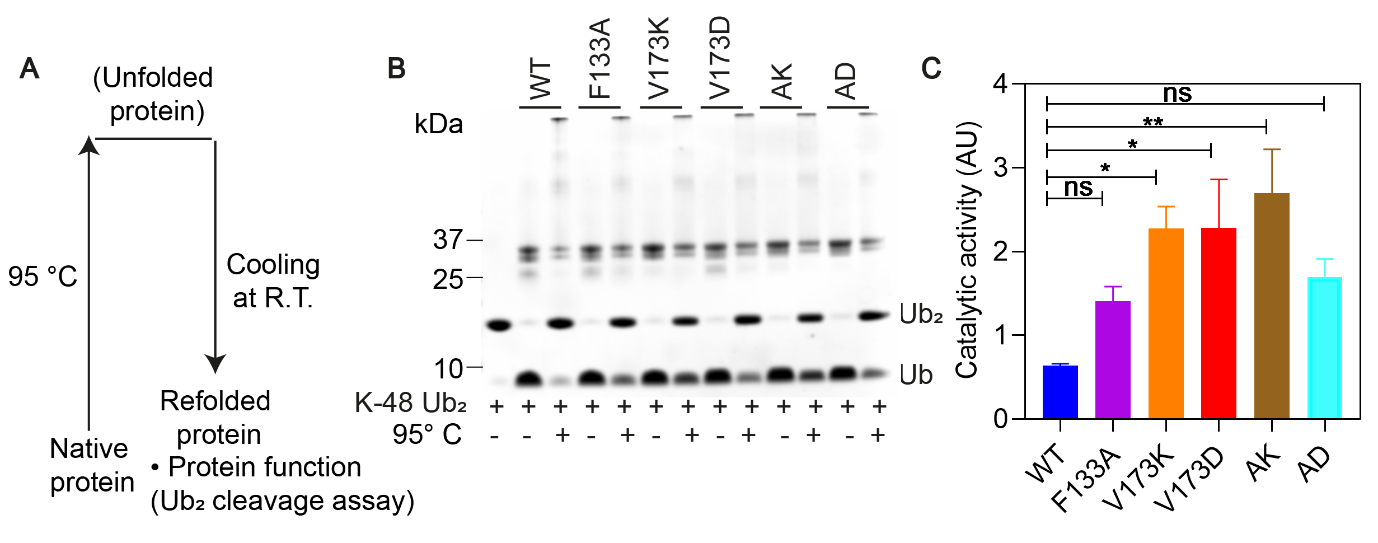


**Figure S5:** **Activity of refolded protein.**

**A:** Experimental workflow of thermal refolding experiment.

**B-C**: Catalytic activity of refolded protein after thermal denaturation was checked by performing diubiquitin cleavage assay. Three independent experiments were carried out and catalytic activity was calculated from the Ub/Ub_2_ ratio and ordinary one-way ANOVA (Dunnett’s multiple comparisons test) was performed to determine the statistical significance. (* corresponds to p-value <0.013, and ** corresponds to p-value 0.0042).


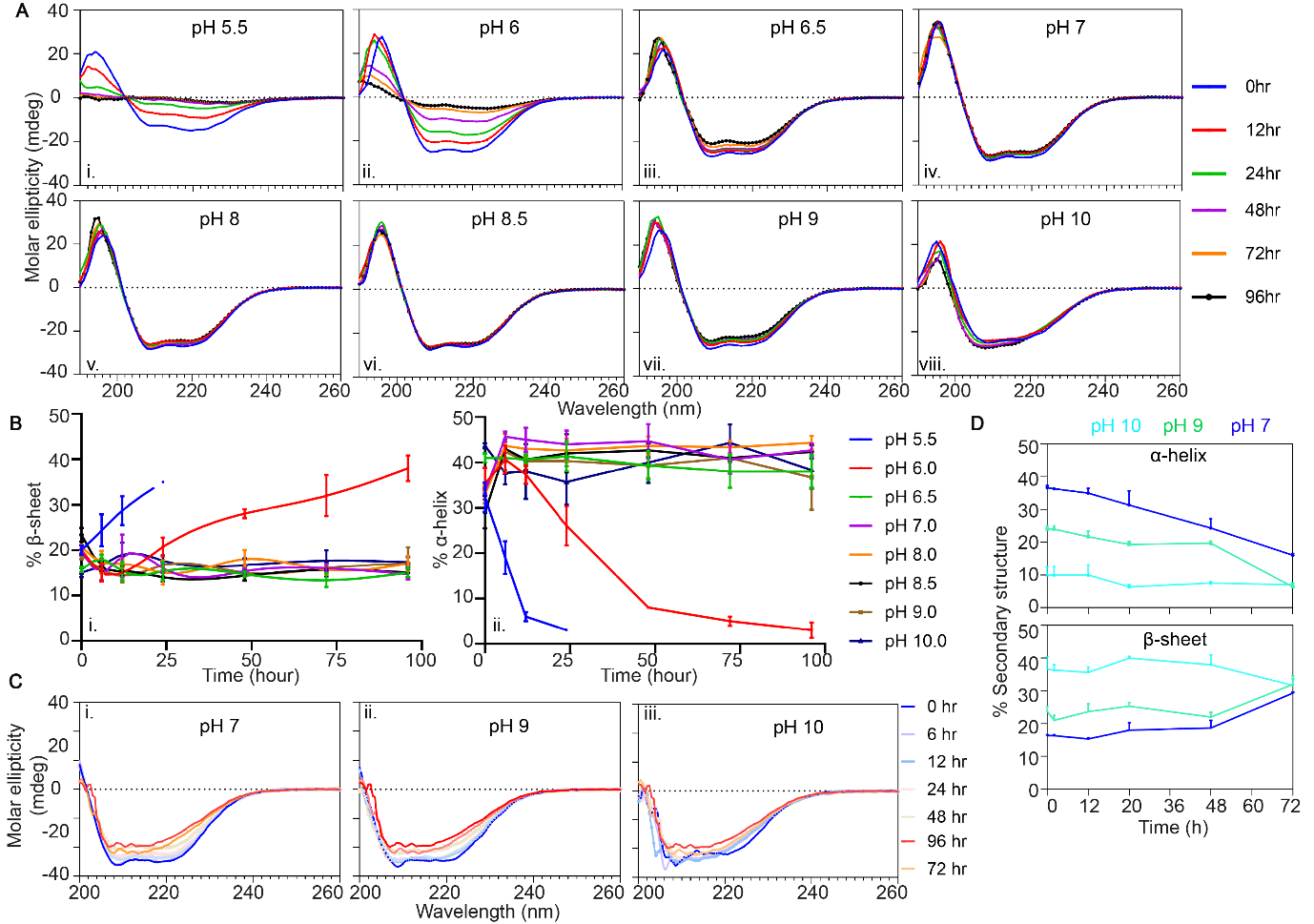


**Figure S6**: **pH-dependent stability of wild-type OTUB1.**

**A**: Time-dependent CD spectrum of wild protein incubated at 25 °C from pH 5.5-10. The mean spectrum of three independent samples was plotted.

**B**: Changes in secondary structure content were calculated using Dichroweb analysis and percent composition was plotted against incubation time.

**C-D**: Time-dependent CD spectrum of wild-protein aggregated at pH 7, 9, and 10. The mean spectrum of three independent samples was plotted (C). Major secondary structure (%) content was quantified using Dichroweb tool from the CD spectroscopy data and plotted by using GraphPad Prism. Error bar represents standard deviation from three independent experiments.


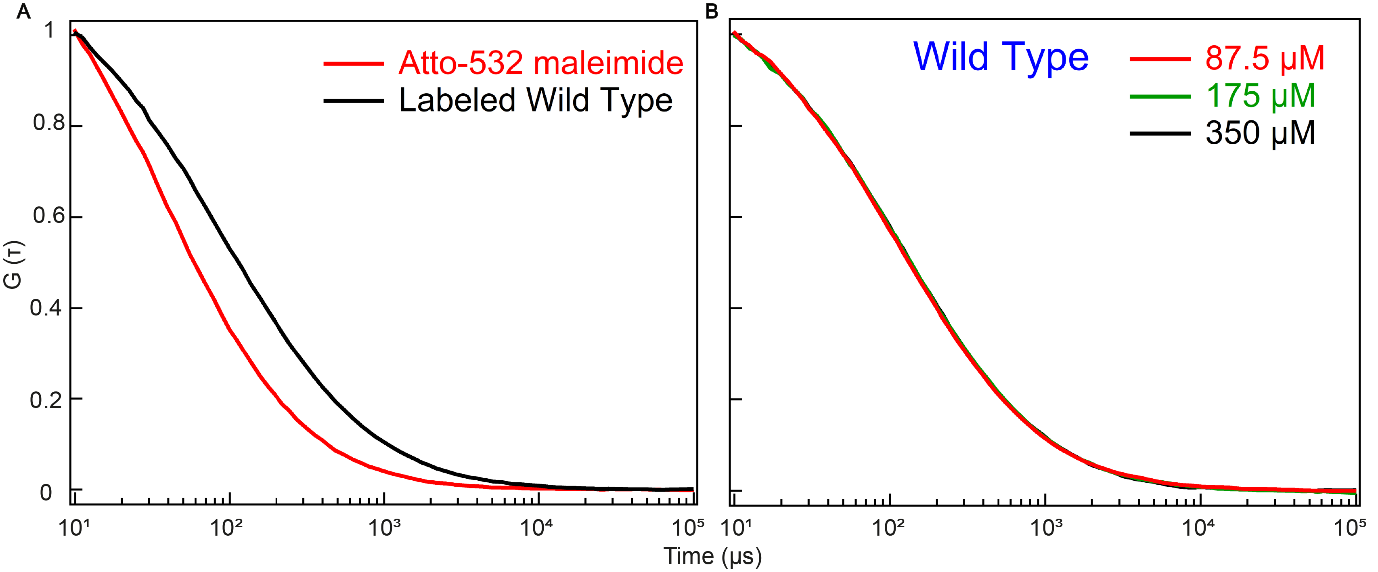


**Figure 7**: **FCS measurement.**

**A:** The correlation function of free dye (Atto-532 maleimide) and dye-labeled wild-type protein.

**B:** Viscosity effect of wild-type protein aggregates- the correlation function at different dilutions shows no difference in the diffusion.


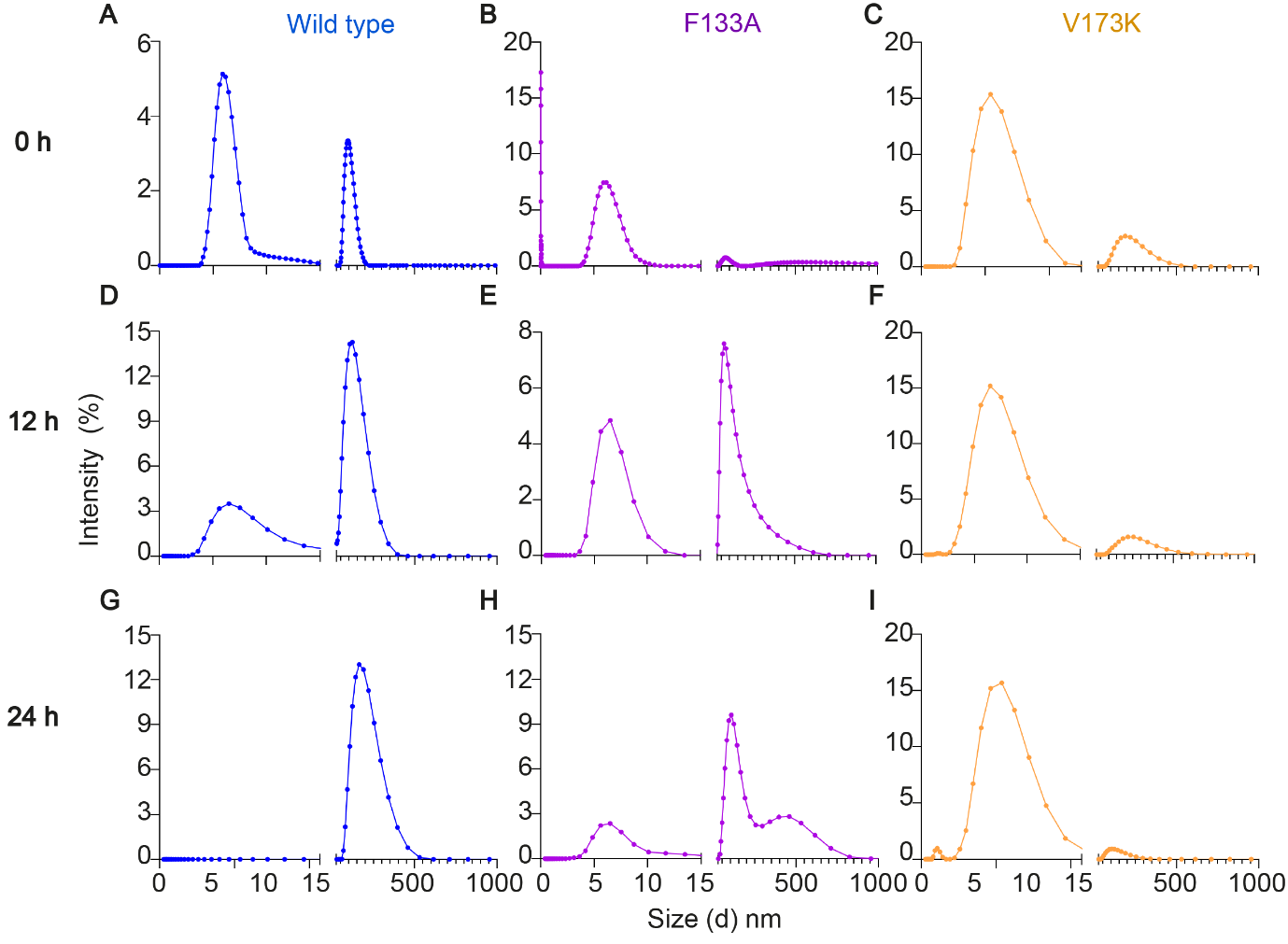


**Figure S8:** **Time-dependent DLS study of OTUB1 aggregates.**

**A-I:** aliquotes of protein aggregates formed at different time intervals (0 h, 12 h, and 24 h) were collected and DLS was performed by using Zetasizer Nano ZS (Malvern instrument). Mean data were acquired from 3 runs, frame averaged over 6 scans, and Gaussian intensity distribution (%) as a function of size (d. nm) was plotted for each protein.


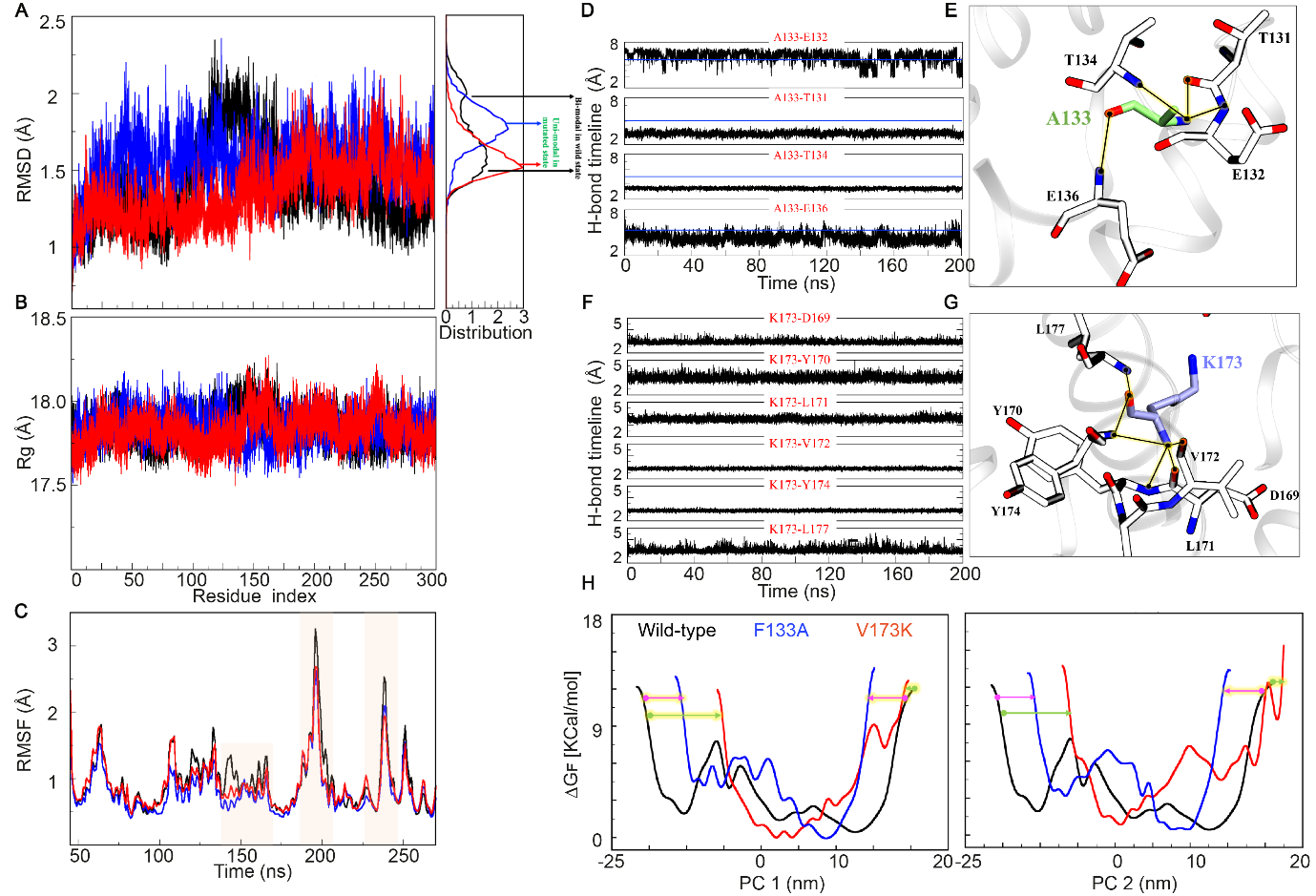


**Figure S9**: **Dynamical characteristics of systems elucidated through MD simulations.**

**A**: Root means square deviation (RMSD) evolution through the course of 300 ns in wild-type and mutated systems are plotted over its backbone atoms and their distribution.

**B**: Changes in Radius of gyration (R_g_) during the course of simulation.

**C**: The Cɑ atomic fluctuation are represented by RMSF-Color coding is followed as black: wild-type; blue; F133A; and red: V173K. The regions showing significant fluctuations are highlighted in transparent bars.

**D-G:** Intra-networking H-bonding interaction of the mutants- (A, B) shows the H-bond networking of A133 and (C, D) represents the H-bond networking of K173 with their neighboring atoms.

**H:** 1D free energy profile of Apo (black), F133A (blue), and V173K (red) systems along PC1 and PC2. The PCs span ranges are shown in nm. The magenta and green arrow represents the divergence of Apo to F133A and Apo to V173K systems.


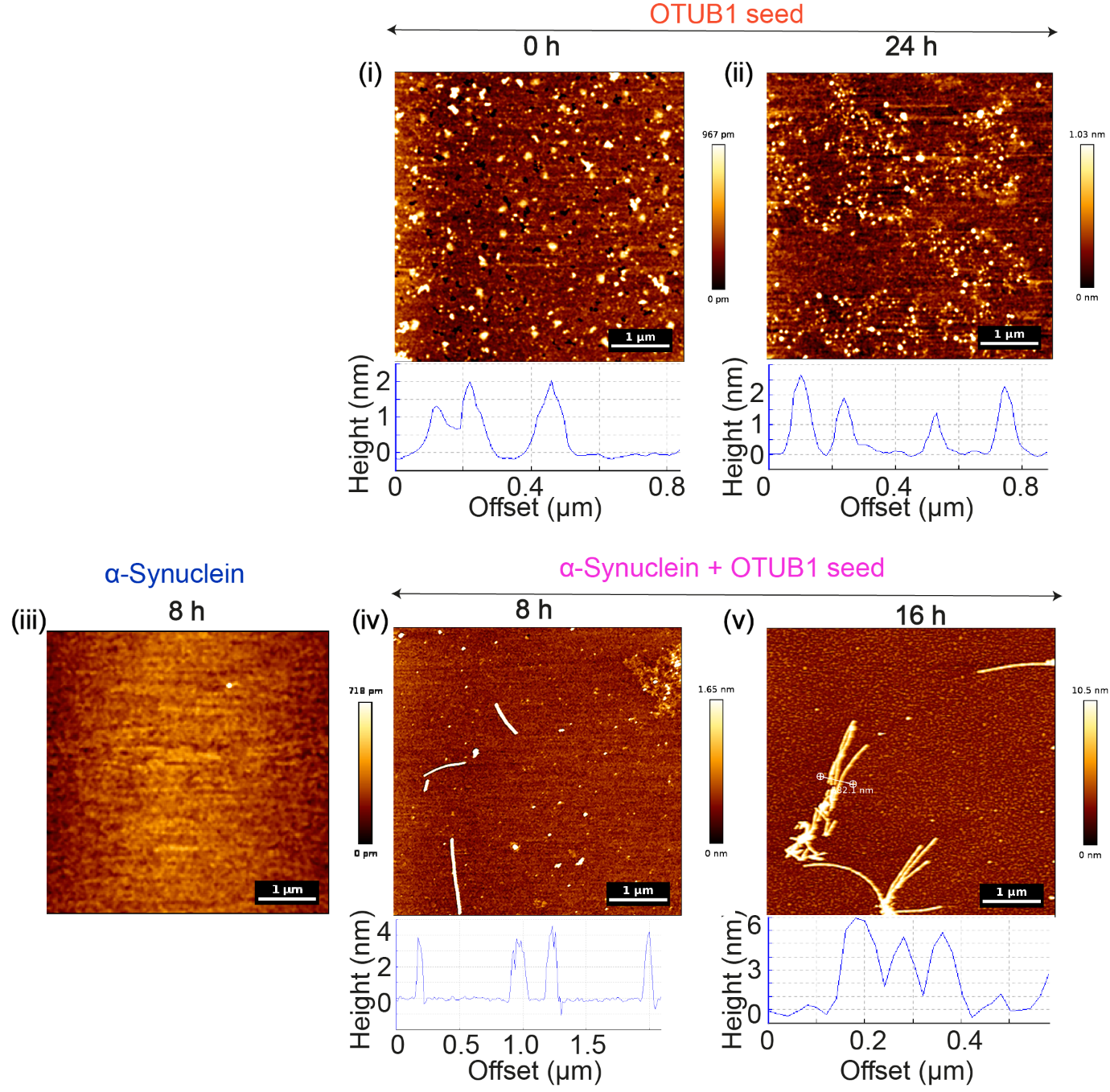


**Figure S10:** **Heterotypic aggregation: in-vitro coaggregation of OTUB1 and α-Synuclein.** Time-dependent Atomic force microscopy (AFM) images showing OTUB1 oligomers as seeds at 0 h (i) and 24 h in aggregating conditions, while α-Synuclein mostly in low molecular weight species in early time points, i.e., 8 h (iii). However, after the addition of wild-type seed, α-Synuclein rapidly undergoes fibrillation (iv-v).

**Table S1: Thermodynamic parameters of OTUB1 and its mutants.**

| **Protein** | **m-value/ slope(kcal/mol.M)** | **[Urea]50% (M)** | **ΔG_D_ (KCal/mol)** |
| --- | --- | --- | --- |
| Wild type | -0.7365 | 4.095 | 3.016 |
| F133A | -0.7964 | 3.765 | 3.291 |
| V173K | -1.416 | 4.927 | 6.979 |
| V173D | -0.768 | 4.133 | 2.891 |

**Table S2: Calculation of diffusion time and hydrodynamic radius of protein aggregates from the FCS correlation data.**

| **Systems** | **Time (h)** | **Peak Diffusion Time,** $\boldsymbol{\tau}_{\boldsymbol{D}_{\boldsymbol{1}}}$**(µs)** | **Peak Diffusion Time,** $\boldsymbol{\tau}_{\boldsymbol{D}_{\boldsymbol{2}}}$ **(µs)** | **Peak Hydrodynamic radius,** $\boldsymbol{R}_{\boldsymbol{h}_{\boldsymbol{1}}}$ **(nm)** | **Peak Hydrodynamic radius,** $\boldsymbol{R}_{\boldsymbol{h}_{\boldsymbol{1}}}$ **(nm)** |
| --- | --- | --- | --- | --- | --- |
| Wild-type | 0 | 157.04 |  | 2.78 |  |
| F133A | 0 | 119.92 |  | 2.12 |  |
| V173K | 0 | 109.61 |  | 1.94 |  |
| Wild-type | 6 | 131.20 | 792.00 | 2.32 | 14.02 |
| F133A | 6 | 119.92 | 505.28 | 2.12 | 8.94 |
| V173K | 6 | 109.61 | 505.28 | 1.94 | 8.94 |
| Wild-type | 12 | 119.92 | 3994.2 | 2.12 | 70.70 |
| F133A | 12 | 131.20 | 1358.2 | 2.32 | 24.04 |
| V173K | 12 | 131.20 | 422.13 | 2.32 | 7.47 |
| Wild-type | 24 | 131.20 | 7493.80 | 2.32 | 132.64 |
| F133A | 24 | 119.92 | 1945.90 | 2.12 | 34.44 |
| V173K | 24 | 119.92 | 422.13 | 2.12 | 7.47 |

**Table S3: Simulation parameters.**

| **ID** | **Systems** | **Time (ns)** | **Total no. of atoms** | **Water molecules** | **Ions** |
| --- | --- | --- | --- | --- | --- |
| 1 | Wild-type | 300 | 32527 | 9622 | 12 Na^+^ |
| 2 | F133A | 300 | 32538 | 9629 | 12 Na^+^ |
| 3 | V173K | 300 | 32535 | 9623 | 11 Na^+^ |

**Table S4: probability of local H-bond network modulation due to the incorporation of mutations.**

**Wild-type:**

| **Donor** | **Acceptor** | **Occupancy** |
| --- | --- | --- |
| GLU176-Main-N | THR175-Main-N | 74.60% |
| GLU176-Main-N | PHE174-Main-O | 56.65% |
| GLU136-Main-N | GLU176-Main-O | 29.01% |
| LYS122-Side-NZ | GLU176-Side-OE2 | 4.135% |
| **PHE133-Main-N** | **GLU176-Main-N** | **16.65%** |
| LYS122-Side-NZ | GLU176-Main-O | 0.12% |
| **GLU176-Main-N** | **PHE133-Main-N** | **6.66%** |
| LYS122-Side-NZ | GLU176-Side-OE1 | 5.00% |
| GLU176-Main-N | GLU176-Side-OE2 | 10.06% |
| GLU176-Main-N | GLU176-Side-OE1 | 11.46% |
| **PHE133-Main-N** | **GLU176-Side-OE2** | **0.30%** |
| **PHE133-Main-N** | **GLU176-Side-OE1** | **0.24%** |

**F133A:**

| **Donor** | **Acceptor** | **Occupancy** |
| --- | --- | --- |
| ALA133-Main-N | THR131-Main-O | 136.72% |
| ALA133-Main-N | GLU132-Main-N | 120.86% |
| ALA133-Main-O | GLU136-Side-N | 67.04% |
| ALA133-Main-N | THR134-Main-N | 55.61% |

**V173K:**

| **Donor** | **Acceptor** | **Occupancy** |
| --- | --- | --- |
| LEU133-Main-N | LYS173-Main-O | 44.69% |
| TYR174-Main-N | LYS173-Main-N | 59.69% |
| LYS173-Main-N | ASP169-Main-O | 154.17% |
| LYS173-Main-N | TYR170-Main-O | 77.74% |
| LYS173-Main-N | VAL172-Main-N | 152.39% |
| LYS173-Main-N | LEU171-Main-O | 62.35% |
